# Establishment and characterization of patient-derived xenograft of a rare pediatric anaplastic pleomorphic xanthoastrocytoma (PXA) bearing a CDC42SE2-BRAF fusion

**DOI:** 10.1038/s41598-023-36107-2

**Published:** 2023-06-06

**Authors:** Nur P. Damayanti, M. Reza Saadatzadeh, Erika Dobrota, Josue D. Ordaz, Barbara J. Bailey, Pankita H. Pandya, Khadijeh Bijangi-Vishehsaraei, Harlan E. Shannon, Anthony Alfonso, Kathy Coy, Melissa Trowbridge, Anthony L. Sinn, Zhong-Yin Zhang, Rosa I. Gallagher, Julia Wulfkuhle, Emanuel Petricoin, Angela M. Richardson, Mark S. Marshall, Alex Lion, Michael J. Ferguson, Karl E. Balsara, Karen E. Pollok

**Affiliations:** 1 Neuro-Oncology Program, Pediatric Neurosurgery, Department of Neurosurgery, Indiana University, Indianapolis, IN 46202 USA; 2 Department of Neurosurgery, Indiana University, Indianapolis, IN 46202 USA; 3 Indiana University Melvin and Bren Simon Comprehensive Cancer Center, Indianapolis, IN 46202 USA; 4 Department of Pediatrics, Herman B Wells Center for Pediatric Research, Indiana University School of Medicine, Indianapolis, IN 46202 USA; 5 Translational Research Integrated Biology Laboratory/Indiana Pediatric Biobank, Riley Children Hospital, Indianapolis, IN 46202 USA; 6 Indiana University School of Medicine, Bloomington, IN USA; 7 Indiana University Simon Comprehensive Cancer Center Preclinical Modeling and Therapeutics Core, Indianapolis, USA; 8 Department of Medicinal Chemistry and Molecular Pharmacology, Purdue University, West Lafayette, Indiana, IN 47907 USA; 9 Center for Applied Proteomics and Molecular Medicine, Institute for Biomedical Innovation, George Mason University, Manassas, VA 20110 USA; 10 Pediatric Cancer Precision Genomics Program, Indiana University School of Medicine, Indianapolis, IN 46202 USA; 11 Division of Pediatric Hematology-Oncology, Indiana University School of Medicine, Indianapolis, IN 46202 USA; 12 Department of Neurosurgery, University of Oklahoma School of Medicine, Oklahoma City, OH 73104 USA

**Keywords:** Histocytochemistry, Proteomics, RNA, Cancer, Surgical oncology, Cancer, Drug discovery, Medical research, Molecular medicine, Neurology, Oncology, Diagnosis

## Abstract

Pleomorphic xanthoastrocytoma (PXA) is a rare subset of primary pediatric glioma with 70% 5-year disease free survival. However, up to 20% of cases present with local recurrence and malignant transformation into more aggressive type anaplastic PXA (AXPA) or glioblastoma. The understanding of disease etiology and mechanisms driving PXA and APXA are limited, and there is no standard of care. Therefore, development of relevant preclinical models to investigate molecular underpinnings of disease and to guide novel therapeutic approaches are of interest. Here, for the first time we established, and characterized a patient-derived xenograft (PDX) from a leptomeningeal spread of a patient with recurrent APXA bearing a novel CDC42SE2-BRAF fusion. An integrated -omics analysis was conducted to assess model fidelity of the genomic, transcriptomic, and proteomic/phosphoproteomic landscapes. A stable xenoline was derived directly from the patient recurrent tumor and maintained in 2D and 3D culture systems. Conserved histology features between the PDX and matched APXA specimen were maintained through serial passages. Whole exome sequencing (WES) demonstrated a high degree of conservation in the genomic landscape between PDX and matched human tumor, including small variants (Pearson’s r = 0.794–0.839) and tumor mutational burden (~ 3 mutations/MB). Large chromosomal variations including chromosomal gains and losses were preserved in PDX. Notably, chromosomal gain in chromosomes 4–9, 17 and 18 and loss in the short arm of chromosome 9 associated with homozygous 9p21.3 deletion involving *CDKN2A/B* locus were identified in both patient tumor and PDX sample. Moreover, chromosomal rearrangement involving 7q34 fusion; CDC42SE-BRAF t (5;7) (q31.1, q34) (5:130,721,239, 7:140,482,820) was identified in the PDX tumor, xenoline and matched human tumor. Transcriptomic profile of the patient’s tumor was retained in PDX (Pearson r = 0.88) and in xenoline (Pearson r = 0.63) as well as preservation of enriched signaling pathways (FDR Adjusted P < 0.05) including MAPK, EGFR and PI3K/AKT pathways. The multi-omics data of (WES, transcriptome, and reverse phase protein array (RPPA) was integrated to deduce potential actionable pathways for treatment (FDR < 0.05) including KEGG01521, KEGG05202, and KEGG05200. Both xenoline and PDX were resistant to the MEK inhibitors trametinib or mirdametinib at clinically relevant doses, recapitulating the patient’s resistance to such treatment in the clinic. This set of APXA models will serve as a preclinical resource for developing novel therapeutic regimens for rare anaplastic PXAs and pediatric high-grade gliomas bearing BRAF fusions.

## Introduction

PXA is a rare benign astrocytic subset of glioma thought to originate from subpial astrocytes or their precursor^1^. The term PXA was first reported in 1979^2^ but not until 1993 was it formally classified as a WHO grade II tumor of the central nervous system (CNS)^3^. The incidence is less than 1% of all astrocytomas with mean age at diagnosis of 29 ± 16 years, but cases as young as 2 and as old as 69 have been reported^4,5^. Disease prognosis is favorable with 3- and 5-years overall survival rate (OS) of > 80% and > 75% respectively^6^. Complete surgical resection is considered the gold standard of treatment, with 82% of patient receiving GTR is associated with 10 year-OS^7,8^. However, this procedure is not always feasible and recurrence of tumor in the same or distant sites has been reported^9,10^. Moreover, an anaplastic type of this disease was identified and recognized as a separate high-grade CNS tumor in 2016, by the WHO^11^. PXA with greater than 5 mitoses/10 high-power field (HPF) were defined as “anaplastic pleomorphic xanthoastrocytoma (APXA)” and classified as WHO grade III^12,13^. In contrast to PXA, APXA tumors exhibit less favorable outcomes with more aggressive clinical presentation, potential CSF spread and leptomeningeal disease;, higher risk of recurrence and hence lower 3 year progression-free and overall survivals (3-year PXA vs. APXA; PFS: 80.2 vs. 32.3% (P = 0.001), OS:87.8 vs. 61,7 (P = 0.184))^14^.

Furthermore, molecular markers that distinguish PXA and APXA have not been established. Frequent whole or partial loss of chromosome 9 with homozygous deletion of CDKN2A/B, recurrent gain on chromosome 7, and *BRAF* mutations such as *V600E* mutations or its chromosomal rearrangement with other genes have emerged as predominant features, each seen in the majority of PXA^15–17^. However, these features can be observed in other glial tumors. For example, APXA and glioblastoma exists on morphologic continuum^11,18,19^. Specifically, epithelioid glioblastoma shares both morphological as well as molecular features with APXA including a high incidence of *BRAF V600E* mutations^18^. On the benign end of the disease spectrum, PXA can also harbor morphologic and molecular similarities to lower grade tumors such as ganglioglioma and pilocytic astrocytoma^20,21^. Gangliogliomas carry the *BRAF V600E* mutation in most cases and have been reported occasionally to contain *CDKN2A/B* homozygous deletions^22^. In addition, pilocytic astrocytoma also carries *BRAF* chromosomal rearrangement resulting in its gene fusion such as KIAA1549-BRAF^23^. Thus, differentiating PXA and APXA from both lower and higher-grade tumors remains difficult due to these morphological and or molecular similarities.

**Figure 1:**
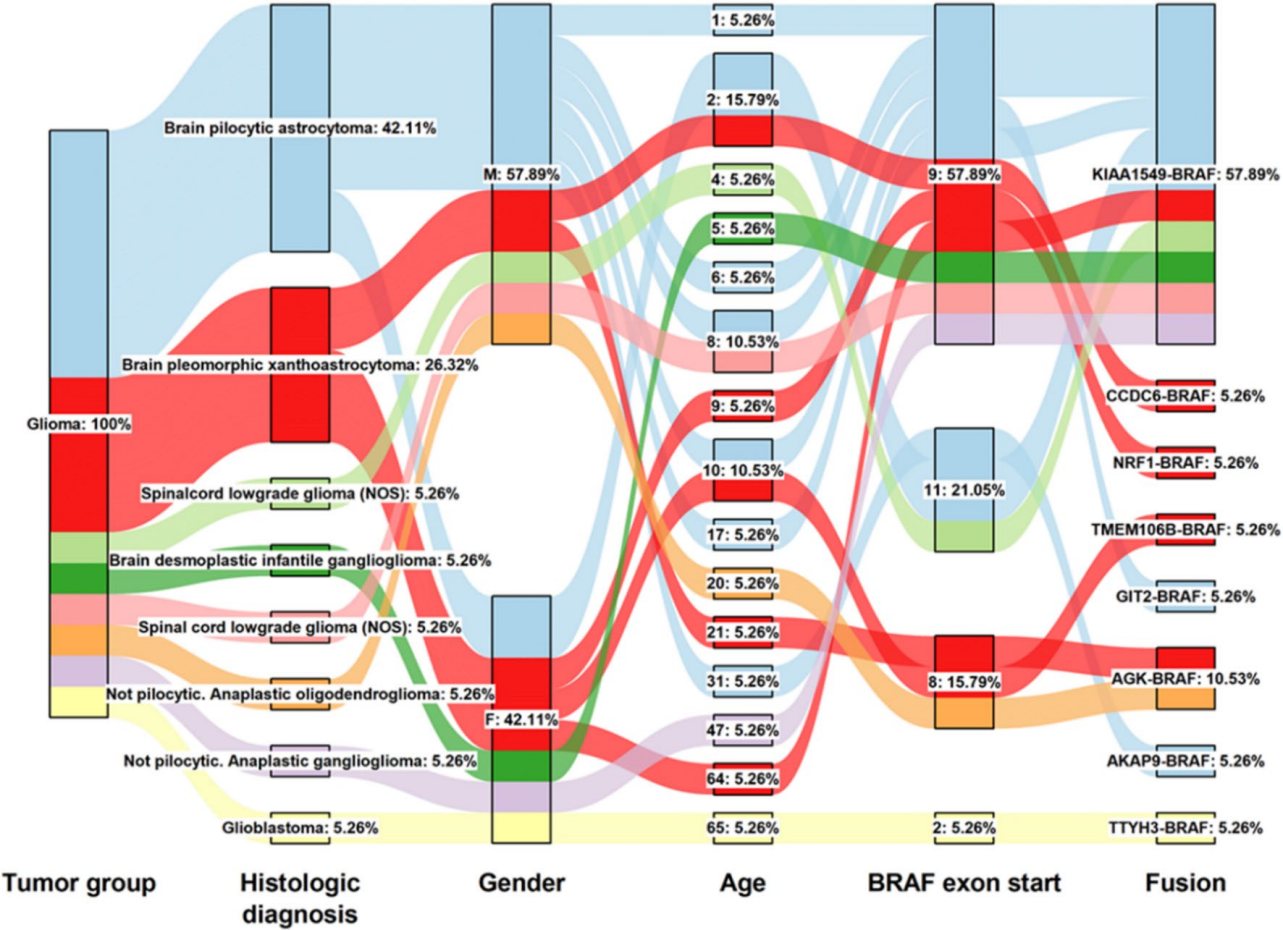
BRAF fusion landscape in glioma. An alluvial diagram of differential BRAF fusion partner across glioma stratified by tumor groups, histologic diagnosis, gender, age, BRAF exon start and fusion partner.

*BRAF* is the most frequently altered gene in the majority of pediatric gliomas including PXA/APXA^24^ with over two-thirds of PXA/APXA harboring alterations in *BRAF.* Among these, BRAF variant V600E and fusion transcripts are the most common variants^15^. *BRAF* fusions themselves are rare driver alterations and represent a different mechanism of BRAF activation compared to other BRAF variants^25^. Recent DNA sequencing of 20,573 solid tumors^25^ identified 60 tumors with in-frame BRAF fusions. Out of these cases (15 tumors groups), BRAF fusions are most identified in glioma (29.5%), melanoma (23%), lung carcinoma (11.5%) and colorectal carcinoma (6.6%) (Supplementary Fig. S1)^4,26,27^. *BRAF* fusions involve intrachromosomal as well as interchromosomal fusion of this gene with a variety of gene partners (Fig. 1). Based on these data BRAF fusion might be one pertinent molecular feature in PXA/APXA malignancy that could serve as a marker for response to BRAF or downstream inhibitors. Furthermore, depending on the data from ongoing clinical trials, *BRAF* fusion screening could become important for clinical decision making in pediatric gliomas^25^. To date, it has been used as an inclusion criterion in 7 clinical trials for malignant solid tumor, of which 7 are open and 0 are closed. In addition, they have been described in a wide variety of malignant neoplasms (Supplementary Fig. S1).

Due to the rarity of APXA, the pathogenesis of recurrence and anaplastic progression is not well understood and there is no current standard therapy. Moreover, the limited number of patients and the young age in most of the patients make clinical trials for this type of brain tumor challenging if not impossible. Therefore, development of clinically relevant models for PXA, especially its anaplastic form APXA, is necessary to gain more insight into this disease.

Patient-derived xenograft (PDX) models are clinically relevant preclinical models widely employed as a strategy to elucidate cancer molecular mechanisms and discovery tools for novel therapeutic testing^26^. In many cases, these models closely resemble the original patient tumors both phenotypically and genotypically. Therefore, PDX models are suitable tools to investigate rare subtypes of pediatric brain tumors where the sample is scarce, since they can be expanded from limited patient tissue and yet still preserve the original tumors’ histological and molecular features. However, fully characterized, and robust preclinical models for both PXA and APXA are limited. To our knowledge, there are currently no commercially available PXA or APXA cell lines and limited existing in vivo models^27^. There is one report of a characterized model of PXA which progressively evolved into APXA^28^, and another two models of PXA were reported with focus on pharmacology testing on models bearing BRAFV600E and non-mutated BRAF^29^. In addition, a PDX model for BRAF fusion is only available for melanoma^30^ and not for PXA/APXA or glioma in general.

In this study, we present a novel APXA PDX model derived from a primary APXA patient with subsequent leptomeningeal spread. Our study provides the first establishment of an APXA model bearing a novel type of BRAF fusion CDC42SE2-BRAF. A comprehensive multi-omics analysis at the DNA, RNA and protein levels is used to compare APXA donor tumor with corresponding PDX. This characterization is complemented by in-vitro and in-vivo functional validation of MEK inhibitor resistance.

### Case description

**Figure 2:**
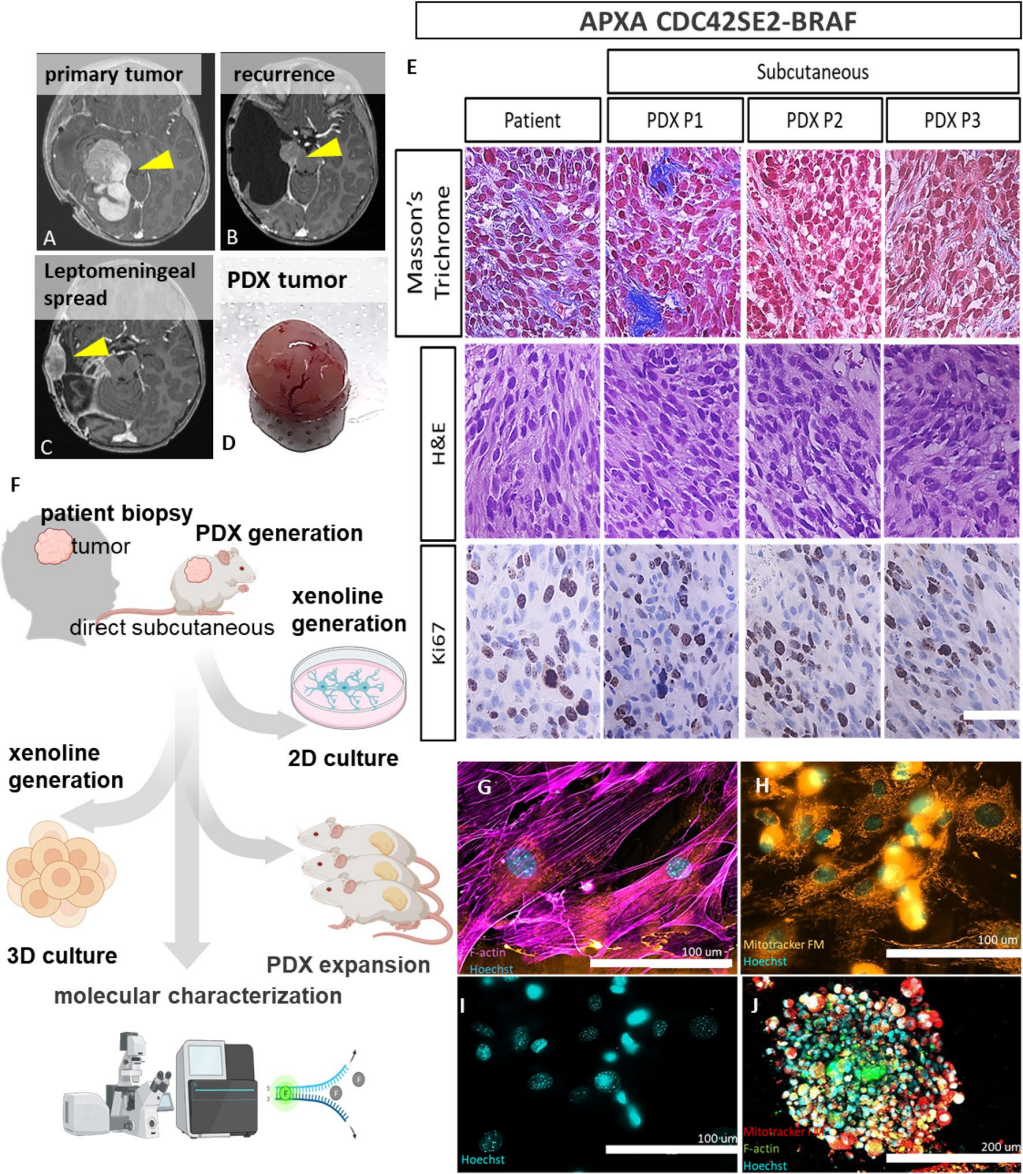
Patient derived models’ generation of anaplastic pleomorphic xanthoastrocytoma (APXA) from leptomeningeal spread. (**A–C**) Patient’s MRI images of (**A**) initial presentation with lesion located in the occipital lobe (yellow arrow), (**B**) recurrence in the primary site (yellow arrow) (**C**) Post-operative images9 months after surgery on (**B**), arrow indicated leptomeningeal infiltration, sample from (**C**) was subsequently implanted for patient derived xenograft (PDX) derivation. (**D**) PDX tumor grown in immunodeficient NSG mice. (**E**) Faithful resemblance of cellular complexity, extracellular matrices and architecture of PDX tumor to the patient tumor represented by Masson’s Trichrome (top), Hematoxylin–Eosin (middle) and proliferative marker Ki67 (bottom). (**F**) Schematic diagram of PDX generation with subsequent expansion followed by integrated omics for molecular characterization purposes; (i) further expansion through serial passaging in NSG mice and (ii) histologic and (iii) molecular characterization. (**G–J**) patient derived xenoline of APXA derived from the same sample in (**C**) stained with: (**G**) cytoskeletal marker, F-Actin, (**H**) mitochondrial activity marker Mito FM, (**I**) DNA double helix marker (Hoechst). (**J**) Confocal image of 3D spheroid culture of xenoline in (**G–I**) stained with F-actin (green), MitoFM (red, and Hoechst (Cyan).

The patient was a developmentally normal 6-year-old male who presented with an acute onset of headache. A CT scan and MRI demonstrated an irregularly enhancing hemorrhagic lesion in the right occipital lobe with surrounding edema, mass effect, and enhancement of the dura (Fig. 2A). He underwent a right craniotomy with gross total tumor resection and pathology was consistent with a diagnosis of an APXA WHO grade III. Genomic testing results were consistent with a homozygous deletion of CDKN2A/B at 9p21.3 and 7q34 fusion; CDC42SE2-BRAF and a remaining wild-type BRAF allele. A local tumor recurrence was found 6 months after first diagnosis (Fig. 2B), and a second resection was performed. Leptomeningeal spread was identified 15 months after initial diagnosis (Fig. 2C) at which the third resection surgery was performed and a tumor specimen for PDX generation was obtained (Fig. 2D). Sections of the right occipital tumor showed a complex glial neoplasm characterized by numerous pleomorphic tumor cells highlighted by a collagen rich region (Fig. 2E). Immunohistochemical (IHC) stains were positive for Ki-67 (Fig. 2E) and negative for BRAFV600E. Therapies with a MAPK family MEK1/2 inhibitor (trametinib), cell cycle family cyclin D1/CDK4 and CDK6 inhibitor, (Ribociclib), and an mTOR inhibitor (everolimus) had no effect on tumor progression and the patient passed away 16 months from initial diagnosis.

### Establishment and characterization of xenografts and cell lines

The pipeline of PDX generation and characterization is depicted in Fig. 2F. Briefly, the resected patient tumor was partitioned into approximately 3 to 5 mm^3^ pieces, processed, and implanted subcutaneously into 8-week-old male NOD/SCID gamma null (NSG) mice. We allowed the tumor implant (PDX RHT128) to grow to a size of approximately 1,500 mm^3^ during the engraftment phase, at which point they were harvested for the following purposes: (i) further expansion through serial passaging in NSG mice and (ii) histologic and (iii) molecular characterization (Fig. 2F) by whole exome sequencing (WES), RNA-seq, and reverse phase protein array (RPPA). To develop the xenolines (Fig. 2G–J), we adopted scaffold free method with protease aided dissociation to culture xenolines in 2D setting (Fig. 2G–I) and scaffold free low surface tension technique for 3D culture (Fig. 2J). Xenolines demonstrated strong cytoskeleton network (Fig. 2G), prominent orthodox shape mitochondria indicating active mitochondrial related metabolism^31^ (Fig. 2H), and paraspeckle rich nucleus^32^ (Fig. 2I) which were associated with differential alternative splicing function and non-coding RNA regulation^32,33^. These observed features in 2D setting are retained in 3D setting (Fig. 2I). PDX tumors were passaged at least 3 times as subcutaneous xenografts with a mean time between passages of 32 days (28–90 days). We then evaluated whether our preclinical models maintained histologic and molecular features.

### Genomic landscape of the original tumor is preserved in PDX passages

**Figure 3:**
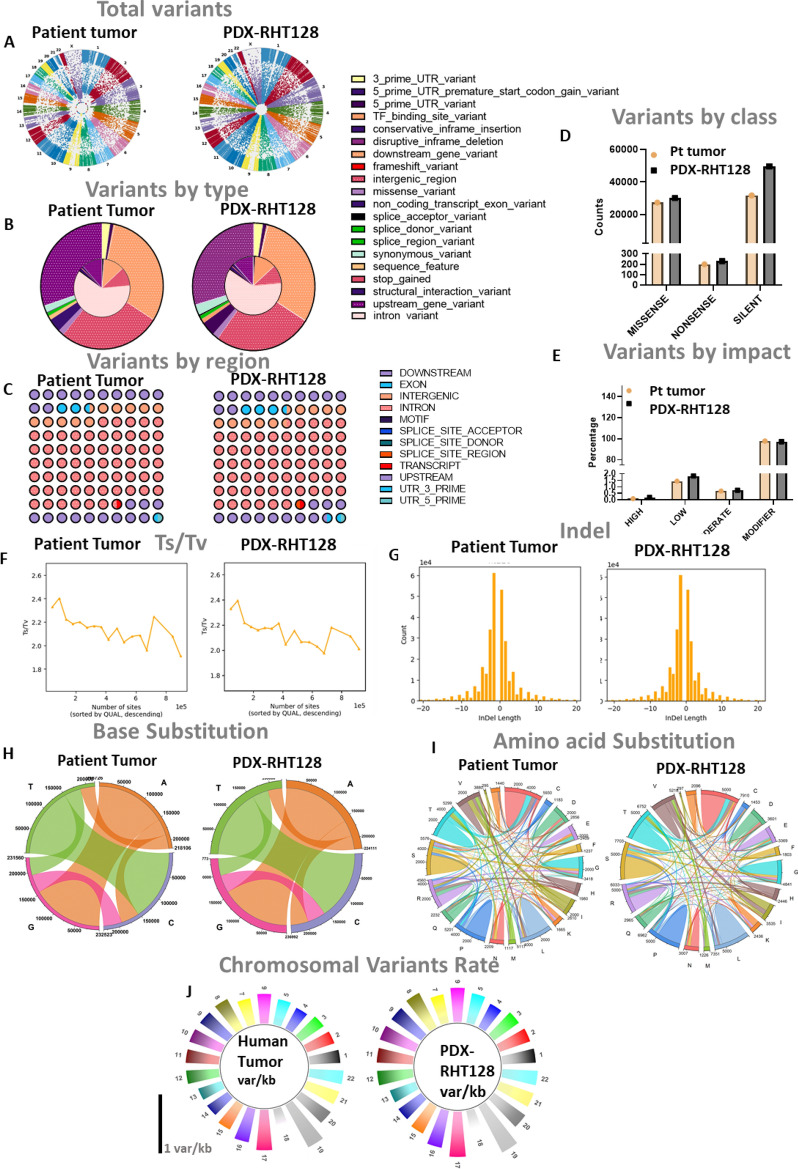
Whole exome sequencing data (WES) reveals genome landscape fidelity between PDX tumor and patient’s tumor. PDX tumor preserves global variants landscape of patient’s tumor: (**A**) circular Manhattan plot of global chromosomal Copy number variation (CNV) and minor allele frequency (MAF), (**B–E**) distribution of annotated variants by: (**B**) type, (**C**) region, (**D**) class, (**E**) impact, (**F**) Exome region of Ts/Tv ratio, (**G**) indel distribution, (**H,I**) Substitution landscape of (**H**) Base, (**I**) Amino Acid, (**J**) Chromosomal variants rate.

Demonstrating concordance between donor and PDX tumor pairs at the molecular level is pertinent to the assessment of PDX tumors as an avatar for primary tumors. In order to demonstrate the genomic fidelity of donor tumor/PDX pairs we analyzed the global genomic landscape encompassing; short variants such as single nucleotide variant (SNV = 1 bp), copy number variation (CNV = 1 kb–100 kb) and structural variation (SV = 100 bp–100 Mb), respectively^34^. WES was performed on the paired original human tumor (patient tumor) and the established PDX (PDX-RHT128) for genome comparisons. The sequencing run generated on average 266–391 MB reads depth. Standard genome analysis tools (GATK) were applied to call genetic variants based on WES sequencing data. After proper variant filtering and annotation, we found that the patient tumor and the PDX-RHT128 indeed shared a similar genomic landscape (Fig. 3A–J). High correlations were observed between samples with Pearson correlation coefficients ranging between 0.794 and 0.839. They shared similar distribution of SNV types (Fig. 3A,B, Pearson’s r = 0.97) and identical exonic as well as intronic mutation distribution (Fig. 3C, Pearson’s r = 0.92). SNV mutation class and impact were then analyzed, and good similarity was observed (Fig. 3D,E). Transition/transversion ratio (Ts/Tv) ranged from 2.135 to 2.4 and 2.14 to 2.4 in patient tumor and PDX sample, respectively (Fig. 3F). Indel distribution was also consistent between patient tumor and PDX sample with indel length range from 1 to 10 bp (Fig. 3G). The subtypes of base substitutions (e.g., C > A, C > G, C > T, T > A, T > C, T > G) were well-preserved in PDXs (Fig. 3H). Amino acids substitutions with the most common substitution for Threonine (T), Valine (V) and Alanine (A) were also retained in PDXs tumor (Fig. 3I). Low mutational burden (Low TMB = 0–5)^35^ was detected in both primary tumor and PDX models (average TMB ~ 3 mutations/Mb). Variant rate per chromosome with average of 4 variants/10kbp were well preserved in PDXs as well as the differential variant rates in each chromosome in PDX tumor (Fig. 3J).

**Figure 4:**
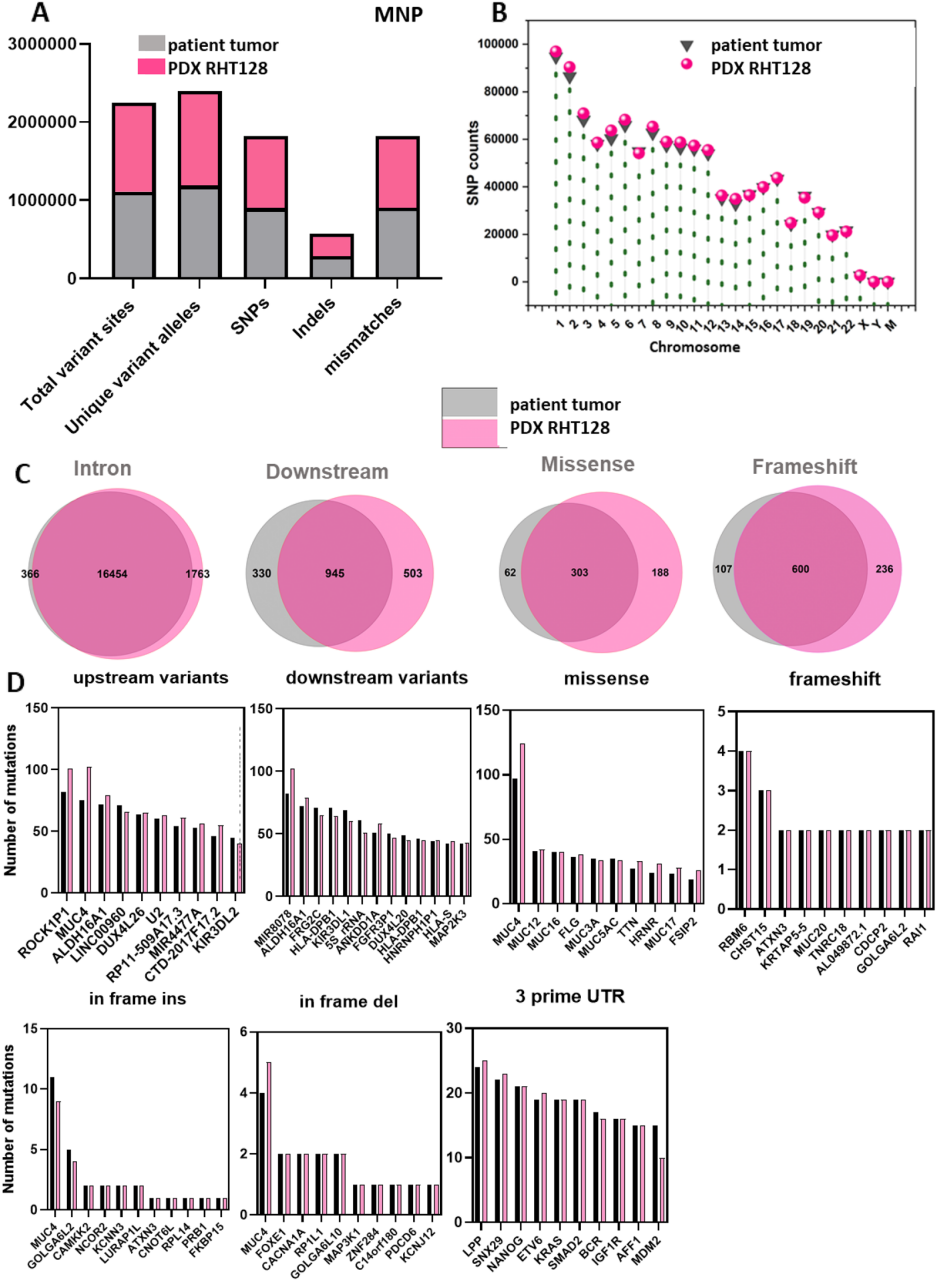
PDX model recapitulates genomic feature of small variants of corresponding patient’s tumor. Preservation of: (**A**) Total number of variants sites, allele, SNP, indel and mismatches (**B**) Chromosome specific total number of SNV in PDX tumor compared to corresponding patient’s tumor, (**C**) Number of preserved small variants by type in PDX tumor: intron, downstream, missense and frameshift, (**D**) Preservation of top genes for select variants in PDX tumor.

Next, we further examined how well short variants identified in the patient’s tumor were reproduced in PDX. The portion of variant sites, unique variant alleles, SNPs, indels and MNPs were well preserved in PDXs tumor (Fig. 4A, Pearson’s r = 0.79). The number of SNPs in each chromosome in patient tumor were reproduced in PDX (Fig. 4B). We also examined overlap in the range of 74%-97% of mutations including intronic mutations, downstream variants, missense, and frameshift between patient and PDX, respectively (Fig. 4C). The genes with most annotated variants in each type of mutation were also consistent between the original patient tumor and PDX (Fig. 4D).

**Figure 5:**
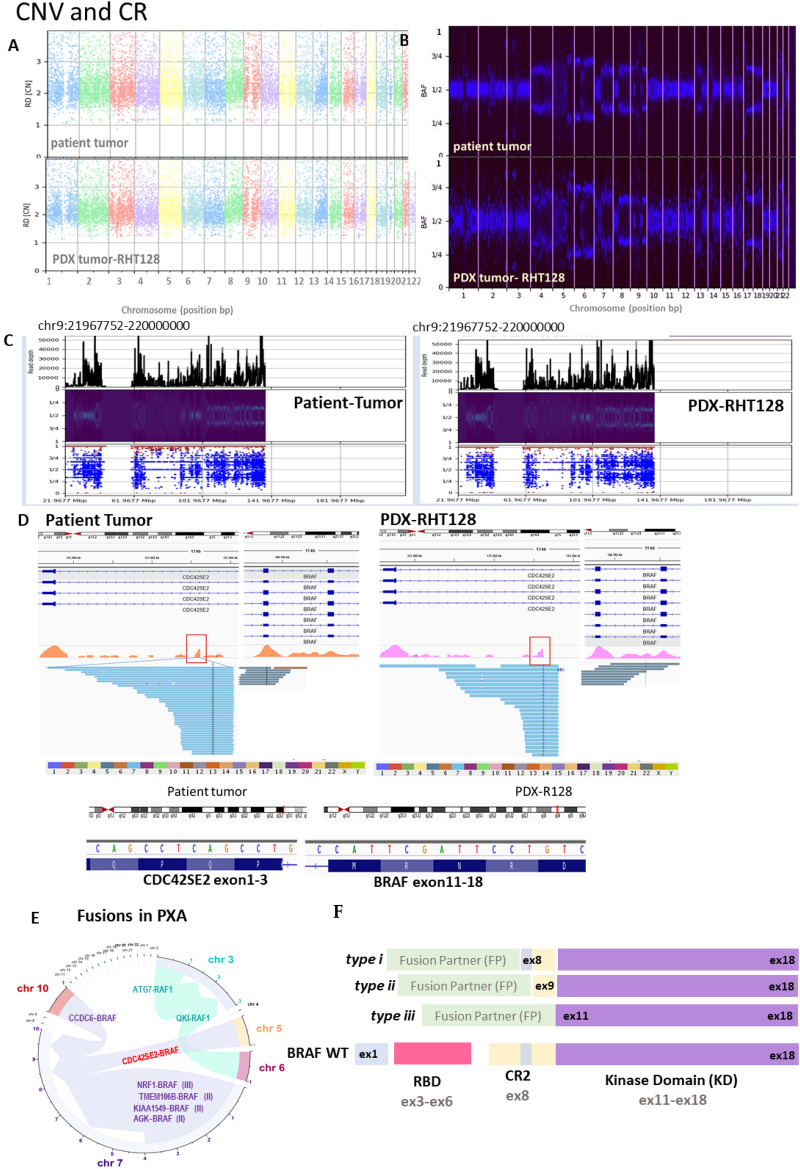
PDX model recapitulates genomic feature of copy number variation (CNV) and chromosomal rearrangement of corresponding patient’s tumor. PDX tumor preserves distribution of CNV in each chromosome, demonstrated by (**A**) Manhattan plot, (**B**) B- Allele frequency (BAF), (**C**) specific homozygous 9p21.3 deletion on CDKN2A/B locus of patient tumor was identified in PDX tumor (coordinate deletion: chr9: chr9:21967752–220000000), (**D**) In-frame CDC42SE2-BRAF fusion was detected in both patient (left) and PDX (right) tumor, with the chromosomal coordinate corresponding to the fusion breakpoint indicated in the red square (GRCh38/hg19) and the fusion breakpoint sequence in blue (bottom), (**E**) Curated gene fusion distribution in PXA mainly consist of interchromosomal and interchromosomal of BRAF or RAF1 gene in 3’ prime position (left), (**F**) Schema of BRAF fusion in the literature based on their protein domain (type I-III) (top, right) and novel BRAF fusion type identified in this work (bottom, right).

Larger structural variants (SV), including duplications, insertions, deletions, inversions and translocations, were observed with marked similarity in genomic structures between parental tumors and PDXs (Fig. 5A–D, Supplementary Fig. S2A–C). Copy number alterations (CNA) were maintained after xenotransplantation (Fig. 5A–C) in each chromosome notably in chromosome 4–9, 17 and 18 (Fig. 5A–C, Supplementary Fig. S2A–C). Consistent with known genomic hallmark of PXA, the loss in short arm of the chromosome 9 associated with homozygous 9p21.3 deletion involving *CDKN2A/B* locus was identified in both the original patient’s tumor and the PDX sample (Fig. 5C). The PDX also retained inter-chromosomal rearrangement between long arms of chromosome 5 and 7 resulting in in-frame fusion of N-terminal CDC42SE3 (exon 1–3) and C-terminal of BRAF (exon 11–18) with breakpoint t (5;7) (q31.1, q34) (5:130,721,239, 7:140,482,820) (Fig. 5D). Preservation of variant allele frequency (VAF) of the fusions was observed between patient’s tumor (VAF patient = 48.4%) and our current model (VAF PDX P1 = 46.3% and VAF PDX P2 = 44.3%), which also indicate the preservation of fusion’s heterozygosity^36^.

Based on a curation of the literature, PXA predominantly harbors intrachromosomal BRAF or RAF1 fusion (Fig. 5E)^25,37–41^ which make this inter-chromosomal fusion novel in this malignancy. In addition, based on exonic breakpoint, there were two types of BRAF fusions commonly found in PXA; type I BRAF retains exon 8–18 of BRAF, indicating conservation of CR2 domain with their autoinhibitory roles^42^, while type II BRAF involves exon 9–18 of BRAF, indicating loss of CR2 domain but retaining portion of some of the autoinhibitory moiety (Fig. 5F)^43^. The CDC42SE2-BRAF fusion, found in both the original patient’s tumor and PDX represent a new type of BRAF fusion (type III), characterized by a complete loss of autoregulatory domain (exon 1–9) but preservation of kinase domain (exon 11–18) (Fig. 5F)^44^. The presence of chimeric fusion RNA transcript was validated via RT-PCR with primers encompassing the fusion junction. (Supplementary Fig. S3).

### Transcriptome and splicing events are maintained in the PDX passages

**Figure 6:**
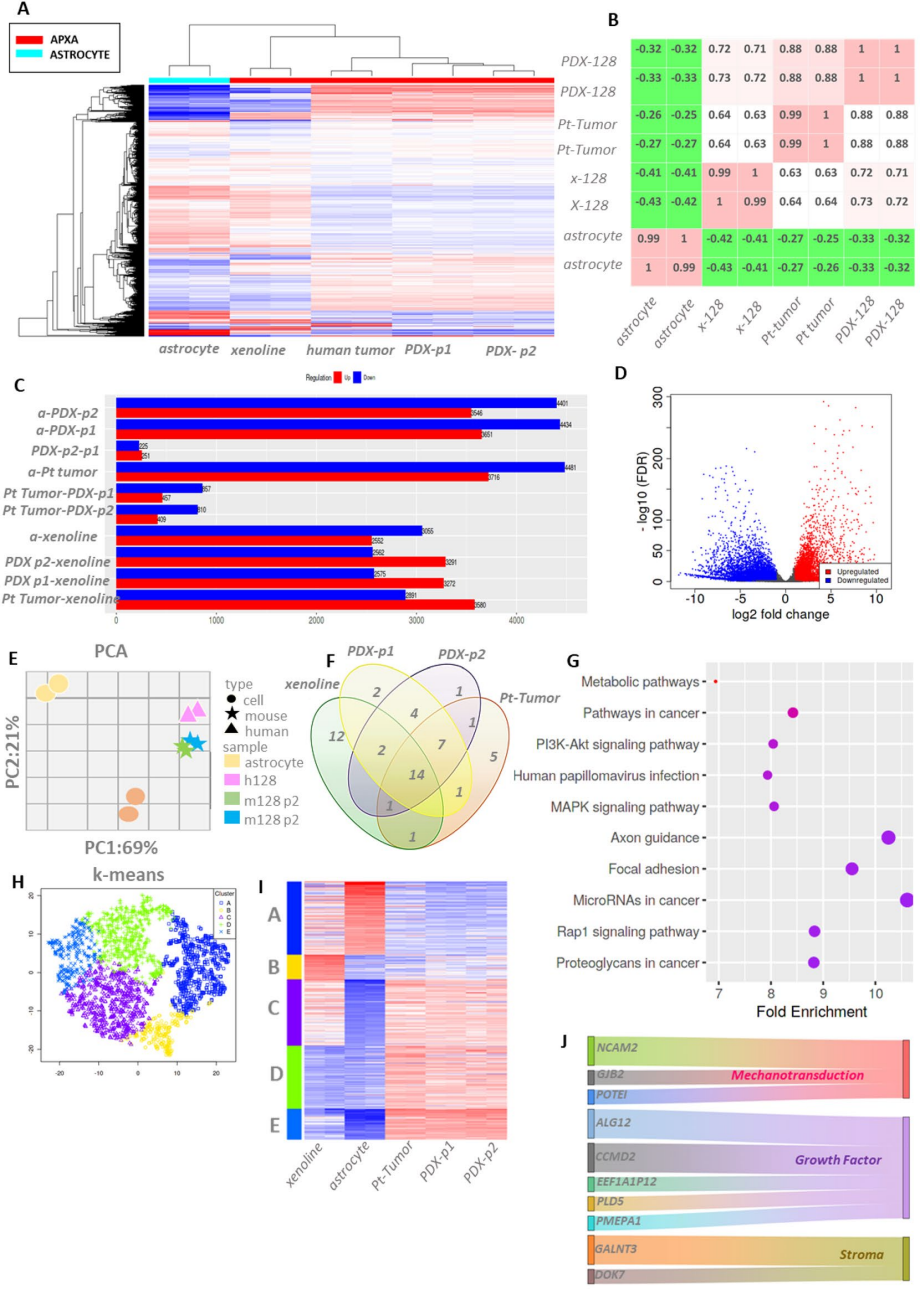
RNA-seq data revealed transcriptomic landscape fidelity between the original patient’s tumor and the PDX. (**A**) Heatmap of gene expression of astrocyte, X-128 xenoline, patient tumor, and PDX tumor passage 1, PDX-RHT128-p1 and passage 2, PDX-RHT128-p2. Color coded panel on top depicts subtype annotation samples astrocyte (cyan) and APXA (red). Source data are provided as a Source Data file. The Spearman correlation was used, and the samples were ordered using hierarchical clustering with complete linkage. (**B**) Sample-sample correlation plot using the top 5000 highest variance genes. (**C**) Differentially expressed genes (DEGs) with false discovery rate (FDR) < 1 and fold change > 2 demonstrate similarity of DEG between PDX and patient tumor, (**D**) Log2 fold change of DEG in APXA vs astrocyte class [red VS cyan in (**A**)], (**E**) Principal component analysis plot showing samples color-coded based on sample name and shape based on sample organism origin, (**F**) Overlap of enriched pathways in each samples type in (**G**) Enriched pathways based on KEGG database based on overrepresentation of DEG in APXA, (**H**) K-means clustering of DEG, (**I**) associated heatmap of K-mean cluster, (**J**) Enriched pathway associated with cluster B and E in K-mean cluster.

To evaluate stability of the expression landscape in PDX passages (PDXRHT128-p1, PDX-RHT128-p2) as well as in the patient-derived xenoline (X-128), a paired end transcriptome sequencing was performed on the matched patient tumor, PDXRHT128-p1, PDX-RHT128-p2, X-128, and normal human astrocytes. Astrocytes were chosen as a reference based on the premise that PXA originates from subpial astrocytes^1,45^. We ranked genes by their standard deviation across all samples and used the top 7500 genes in unsupervised hierarchical clustering. Initial clustering using these top variable genes separated the patient tumor, PDXs, and xenoline from astrocytes, driven by a gene set that is predominantly expressed in the patient tumor and PDXs (Fig. 6A). Variations among PDX passages as well as between PDX and matched human tumors are minimal. Lower similarity is observed between xenoline X-128 and patient tumor as well as xenoline and PDX tumors. These observations can also be confirmed by the correlation matrix (Fig. 6B), showing higher correlation between patient tumor, and PDXs(r = 0.88) than xenoline to patient tumor (r = 0.63). Negative correlation between astrocyte and PDX as well as with matched tumor were noted. Differential expressed gene analysis between samples normalized to astrocytes revealed genes displaying log2-fold higher expression in PDXs samples, matched patient tumor and xenolines (patient tumor = 4481, PDX = 4401, xenoline = 3055, respectively) (Fig. 6C,D). Some divergence in differentially expressed genes between PDX passages as well as PDX-patient tumor pair were observed (PDX passage p1-p2 = 225, PDX-patient tumor = 457, respectively). Concordantly, principal component analysis (PCA) using first and second principal explaining 69% and 21% variance demonstrated patient tumor–PDX pairs and xenolines clustered adjacent to one another (Fig. 6E) further supporting fidelity of the gene expression landscape upon xenotransplantation. Further pathway analysis using KEGG database^46,47^ of differentially expressed genes revealed conservation of enriched pathways (FDR < 0.1, P < 0.05) in patient tumor, PDXs and xenoline (Fig. 6F,G). The top enriched pathways include cancer-associated metabolic pathways, PI3K-AKT, MAPK, FAK, oncogenic miRNA, RAP1 and proteoglycan (Fig. 6G).

**Figure 7:**
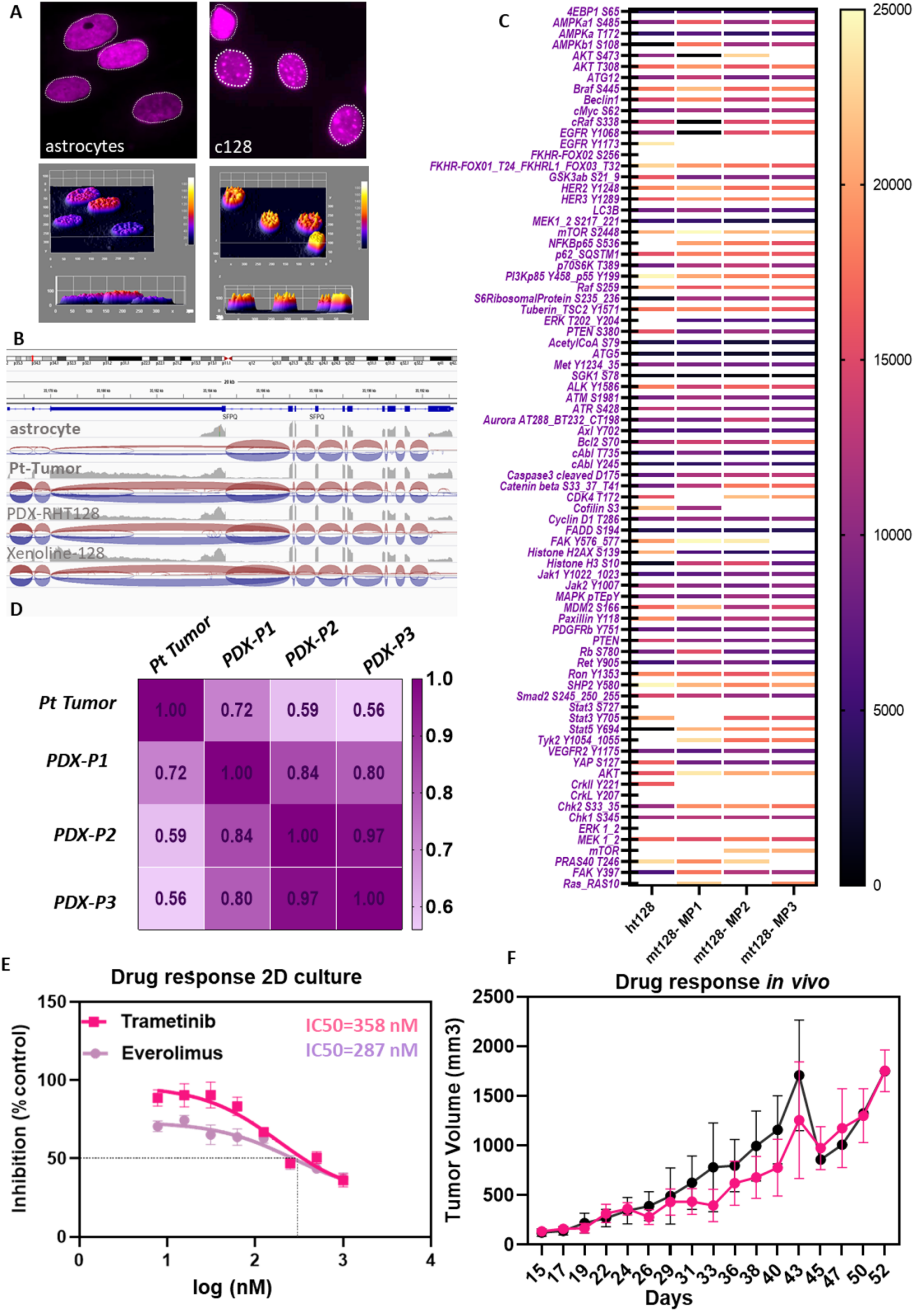
Phenotype signature and conservation of phosphoproteomic landscape, splicing activity and therapeutic response in PDX. (**A**) Identification of the presence of nuclear speckle localization in APXA xenoline compared to normal astrocyte (top), three-dimensional intensity mapping validates the paraspeckle occupancy in APXA (bottom), (**B**) Splice junction sashimi plot identified preservation of differential splicing landscape of paraspeckle component gene, SFPQ, in PDX, xenoline, and patient tumor, compared to astrocyte. (**C**) Heatmap RPPA data, demonstrating conservation of total protein and phosphoprotein of selected kinases in PDX passage 1, 2 and 3 compared to corresponding patient’s tumor. (**D**) Sample-sample correlation matrices based on RPPA data. PDX and xenoline retain drug resistance as the parental patient’s tumor. (E) in vitro drug insensitivity of APXA xenolines in response to the MEK 1/2 inhibitors; Trametinib, and mirdametinib (PD0325901) and mTOR inhibitor Everolimus, (**F**) in vivo drug response graphs depict the average tumor volume (mm^3^) for treatment groups (MEK inhibitor, mirdametinib, magenta) compared to the vehicle control group (black) at each measurement point, with standard deviations plotted (*n* = 5–8 mice/group). Treated group is not significantly different from untreated controls at any point of measurement (ANOVA; P > 0.05).

Noteworthy, the xenoline slightly diverged from the patient tumor -PDX cluster in the PCA coordinate. To interrogate the difference between the patient tumor -PDX pair and patient tumor-xenoline pair, we performed k-means clustering to identify whether gene expression signatures exist in the xenoline allowing separation into a distinct group compared to the patient tumor-PDX pair. Stable clustering was achieved at k = 5 where k denotes the number of clusters. Genes deemed as key drivers of the clustering (meta genes) are listed in Supplementary Tables S1–S5. The signature of genes responsible for driving the separation of xenoline from PDX-tumor pairs were then identified (Fig. 6H,I, Cluster B, yellow subset). Further gene ontology analysis^48^ of these genes reveals clusters encompassing strong functional themes associated with mechanotransduction, growth factor, and stroma interaction (Fig. 6J). Utilizing the X-128 xenoline to study cell biology of APXA, we observed a differential phenotype in the xenoline cells compared to astrocyte cells, notably its prominent expression of the nuclear paraspeckle component (Fig. 7A). Further analysis of the RNA transcript splice junction^49^ revealed a differential splicing event of the gene responsible for paraspeckle complex assembly SFPQ^32^ (Fig. 7B), which was preserved in the PDX passages and the X-128 xenoline (Fig. 7B).

### Proteomic and phosphoproteomic landscapes are retained in PDX passages

To further interrogate conservation of the expression landscape at the protein and signaling levels, patient tumor and PDX passages (P1-3)-were subject to Reverse Phase Protein Array (RPPA)^50^ anlaysis against total (n = 5) and phosphorylated (n = 75) proteins. Unsupervised hierarchical clustering on differential expression analysis based on protein expression and phosphorylation status confirmed high correlation average (r2 = − 0.702) of PDX-human tumor pairs at the protein and post translational modification (PTM) level (Fig. 7C,D). Overall, the enrichment of protein expression and PTM was maintained through serial passaging of the PDX (Fig. 7C,D). Top enriched proteins and PTMs in PDX-human tumor pairs include: ERK1, ERK2, ERKpT202, ERKpY204, RAS10, STAT3 Ser727, CrkL Y207, MTOR, PTEN, NFKBp65, S536, FKHR-FOXO2 S256, and PI3Kp85 Y458.

### Drug sensitivity of the patient tumor is evident in the X-128 xenoline and PDX RHT128

The fidelity of the anti-tumor response in the PDX and xenoline models was systemically evaluated using the MEK inhibitor trametinib and mTOR inhibitor Everolimus, both of which were previously used in the patient. In addition, the MEK inhibitor Mirdametinib which has a more favorable blood brain barrier (BBB) profile compared to trametinib^51^ was also evaluated. Drug inhibitory analysis in monolayer X-128 revealed higher IC50 values for MEK inhibitors and mTOR inhibitors (Fig. 7E, trametinib IC50 = 768 nM, mirdametinib IC50 = 5996 nM, everolimus IC50 = 274 nM) relative to IC50 values insensitive cells which are in picomolar to single digit nanomolar range, e.g., such as trametinib in HT-29 (IC50 = 0.48 nM) and COLO205 (IC50 = 0.52 nM)^52,53^, mirdametinib in TPC-1 (IC50 = 11 nM) and K2 (IC50 = 6.3 nM)^54^, and everolimus in MDA-MB-468 (IC50 = 1 nM) and BT549 (IC50 = 1 nM)^55^.

MEK inhibitor monotherapy with mirdametinib, was conducted in the PDX RHT128 using a 43-day dosing regimen. No significant differences in PDX-RHT128 tumor volume (p > 0.999) over time were observed at any point of measurement between vehicle group and treatment groups (Fig. 7F). Notably, the dose used in our in vivo study (10 mg/kg) is tenfold higher than the dose of 2 mg/m^2^ body surface area (BSA) used in a recent clinical trial with mirdametinib^56^. The human dose in the clinic equates to ~ 1 mg/kg in mice according to Dubois^57^ and Mosteller^58^ formulation.

### Integrative omics reveal potential actionable pathways

**Figure 8:**
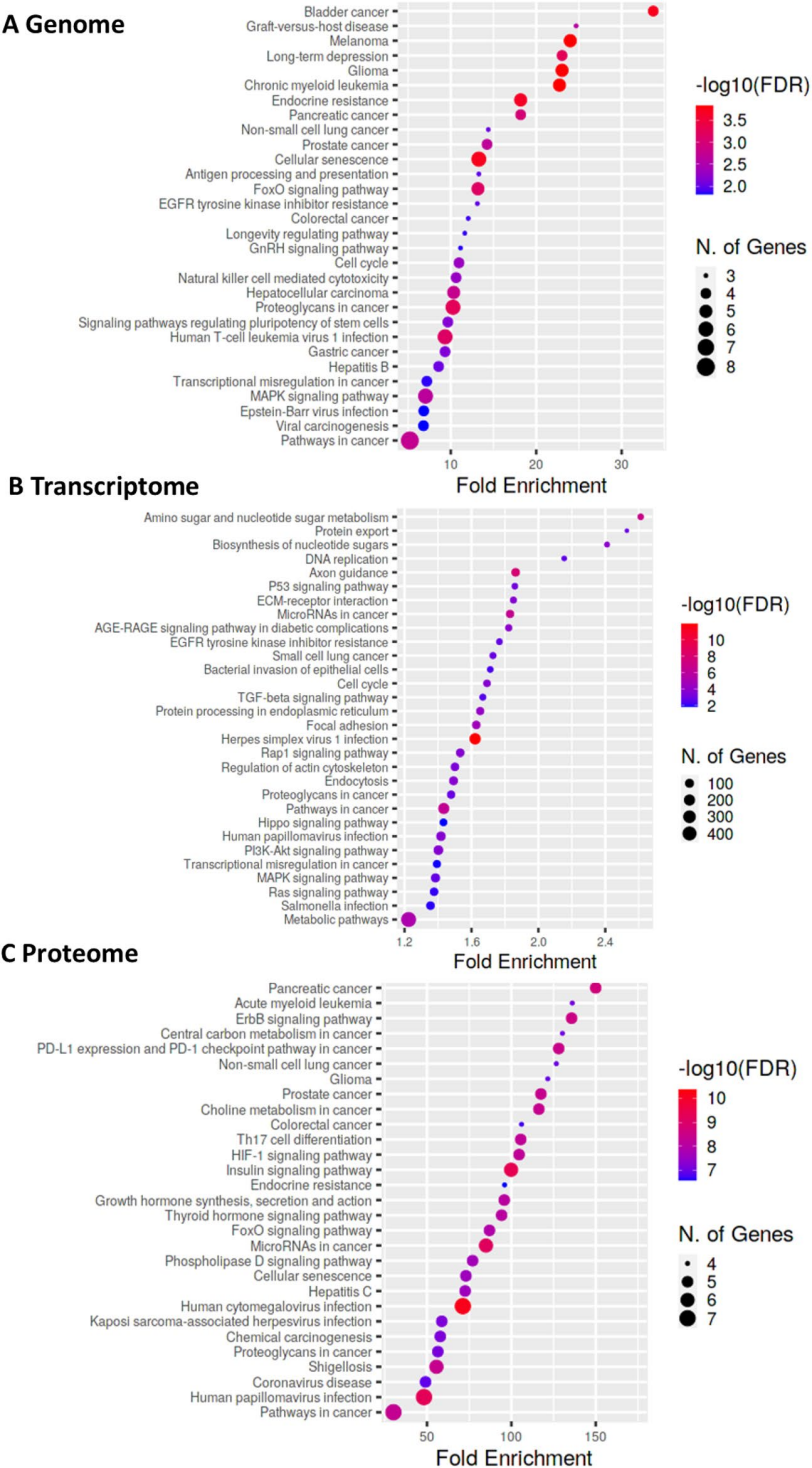
Integrative omics for identifying potential actionable pathway.

To evaluate the utility of the APXA profiled models in the identification of therapies for this difficult-to-treat pediatric tumor, actionability was assessed across WGS, RNA-seq, and RPPA. Pathway analysis based on KEGG database on top altered genes in DNA, RNA as well as at the protein and PTM level revealed distinct potential actionable pathways (FDR < 0.1) in each landscape (Fig. 8A–C). Further analysis on each of the top 30 pathways in each -omics landscape (DNA, RNA, and protein) demonstrated overlap of actionable pathways among the landscapes (Supplementary Fig. S4). Between genome and transcriptome landscape, three actionable pathways were identified: Cell cycle (KEGG04110), transcriptional misregulation in cancer (KEGG05202) and MAPK signaling pathway (KEGG04010). Two actionable pathways were shared between transcriptome and proteome landscape: miRNA dysregulation in cancer (KEGG05206) and human papillomavirus infection (KEGG05165). Interestingly, a higher number of overlap pathways was noted between the DNA and protein landscapes encompassing eight pathways: Glioma (KEGG05214), Endocrine resistance (KEGG01522), Pancreatic cancer (KEGG05212), Non-small cell lung cancer (KEGG05223), Prostate cancer (KEGG05215), Cellular senescence (KEGG04218), FoxO signaling pathway (KEGG04068), and Colorectal cancer (KEGG05210). Furthermore, overlap among DNA, RNA and protein landscapes were also identified, including EGFR tyrosine kinase inhibitor resistance (KEGG01521, Supplementary Fig. S5A), Proteoglycans in cancer (KEGG05202, Supplementary Fig. S5B) and Pathways in cancer (KEGG05200, Supplementary Fig. S5C).

## Discussion

Brain tumors are collectively the most common solid tumor in children comprising heterogenous subtypes with differential prognoses. Despite the tremendous progress that has been made in therapeutic management leading to improved outcomes, there are subsets of patients who remain particularly difficult to treat. These subsets with recalcitrant traits are usually rare thus the understanding of the molecular underpinnings of these tumors is limited, standard treatment course is lacking, and enrollment in clinical trial is challenging. PXA is a low-grade subset of pediatric glioma, the most common type of pediatric brain tumor, with surgical resection being curative. Unfortunately, complete surgical resection in children is not always clinically feasible. Local or distant disease recurrence is highly plausible in the long-term management of this disease, Anaplastic PXA, can result from progression of an initially low-grade PXA into more aggressive anaplastic form (WHO III). However, some patients, as is the case here, present with APXA at the initial diagnosis. Despite the low incidence of PXA and APXA, the true incidence of these subtypes may be well underestimated, due to their shared features, resulting in diagnostic challenges, with higher grade glioma, glioblastoma, and lower grade gliomas such as pilocytic astrocytoma and ganglioglioma.

Developing avatars for PXA and APXA will be critical to gain more insight into mechanisms of disease progression and develop effective and safe therapeutic regimens for this recalcitrant disease. To date, preclinical models for PXA and APXA are very limited. There are no commercial cell lines available and only two published studies of a characterized PXA including PXA that progressed to APXA by serial passaging in immunodeficient mice^28^, and another PXA model with BRAFV600E mutation^29^. The primary APXA developed in our study is derived from the leptomeningeal spread bearing a CDC42SE2-BRAF fusion and one wildtype BRAF allele. Leptomeningeal disease, which is often present with APXA, is a malignancy characterized by dissemination of cancer cells to the cerebrospinal fluid (CFS) where APXA cells can travel to a distant site from the original tumor site. To the best of our knowledge, this is a novel fusion in APXA. However, similar fusion with different breakpoint has been reported in a low-grade malignant soft tissue tumour^59^. Noteworthy, CDC42SE2-BRAF fusion identified in our APXA model is a novel type of BRAF fusion, in which BRAF has completely lost its RAS binding domain and autoinhibitory domain while retaining its kinase domain. This fusion itself serves as a unique marker of this APXA tumor and is maintained in the APXA PDX-RHT128 passages (MP1-MP3) and the xenoline X-128. Moreover, in addition to APXA being rare, next generation sequencing is not routinely conducted in this disease. Therefore, there is a possibility that other cases of APXA with this gene fusion have gone undocumented. Thus, we hope this work will serve as a precursor for other future work in APXA to report and document the structural rearrangement which will help to further delineate the diagnostic or therapeutic significance of this fusion. Studies focused on the functional impact of the CDC42SE2-BRAF fusion are in progress. *BRAF* is reportedly the most altered gene in pediatric glioma, including BRAF fusion alterations. Therefore, our PDX and xenoline will not only contribute to the limited list of models for PXA/APXA but also to the growing list of avatars for pediatric glioma in general.

A major goal in the field of preclinical modeling is to develop models that mimic patient tumor with sufficient fidelity to enable therapeutic discovery. However, methods to measure the extent to which a cancer model resembles or deviates from the original patient tumor are diverse, and there are currently no accepted standardized analytical pipelines. Most studies involving PDX generation usually rely on histological comparisons and focus on specific molecular changes such as mutation or copy number alteration signatures. Furthermore, molecular characterization usually is performed on a single specific landscape such as the genome or transcriptome only. In this work we characterized the fidelity of the APXA PDX-RHT128 to its original patient tumor with a comprehensive approach involving histology characterization and molecular evaluation at the DNA, RNA and protein levels. We believe that the faithful recapitulation of each molecular landscape is complementary to each other, improving these target identification and validation, that is critical for translation of basic discovery to clinical application. In this work, variations across PDX passages as well as between PDX and matched human tumors were minimal. Our data are consistent with the retention of the patient’s molecular signatures at the DNA, RNA and protein levels, most notably structural variation including chromosome 9 homozygous loss on *CDKN2A/2B* locus, *CDC42SE2-BRAF* fusion, differential transcriptomic landscape compared to non-malignant astrocytes, enriched oncogenic pathways such as MAPK, PI3K AKT and Focal adhesion pathway, as well as highly expressed proteins and PTMs involved in major oncogenic pathways. Molecular characteristics are preserved in PDX serial passages as well as in the X-128 xenoline. There were slight differences in fidelity between xenoline and PDX. According to our multi-omics analysis, the discrepancy between xenoline and PDX can be attributed to diverse microenvironment and nutrient source requirements between the PDX and the xenoline. In addition, based on our analyses of the DNA, RNA, and protein landscape, we deduced potential actionable pathways for APXA. Each analysis landscape suggested similar and different targetable pathways, supporting the notion that comprehensive molecular characterization on different landscapes will provide complementary information. For instance, overlapping enriched pathways between genomic and transcriptomic data suggest actionable pathways of MAPK, cell cycle and transcriptional dysregulation. Two of these pathways were targeted in this patient using MAPK pathway inhibitor trametinib and cell cycle inhibitor Ribociclib; however, the tumor continued to progress. Drug sensitivity of our model upon xenotransplantation and xenoline derivation also recapitulated the MEK inhibitor resistance observed in the patient clinical response. We used the MEK inhibitors trametinib and mirdametinib in the in-vitro drug sensitivity testing adopting clinical management of patient. However, for in-vivo testing we opted to use mirdametinib for its superior BBB properties^56^. Interestingly, when we include top molecular alterations at the proteomic level, possible alternate pathways are evident such as EGFR tyrosine kinase inhibitor resistance, proteoglycans in cancer, and other pathways in cancer providing opportunities to explore a multi-agent approach for this disease.

In summary, this characterized APXA CDC42SE2-BRAF fusion model will be a valuable tool for investigations in the rare brain tumor scientific community. Our data highlight practical applications of integrative—omics including genomic, transcriptomic, and proteomic/phosphoproteomic analysis to assess model fidelity as well as maximize predictive capacity. The PDX and xenoline models will now allow for testing of strategic combination therapies in both ectopic and orthotopic APXA modeling approaches respectively. However, to ensure successful engraftment with limited sample size, we did not attempt to directly implant patient tumor biopsy intracranially. We elected to use the direct implant of the APXA tumor specimen without in-vitro manipulation prior to injection. In our PDX pipeline of rare pediatric and AYA tumors, we have found maintenance of -OMICs signature in a variety of other tumors using this implant method (Pandya et al., Cancers 2022). This holds true for this model as well. The -omics landscape at the DNA, RNA, and protein levels was maintained for the oncogenic-associated pathways in the APXA models (PDX and xenoline) compared to the biopsy from which it was derived. Therefore, our current subcutaneous model lacks the relevant tumor microenvironment, which prevents our ability to assess impact of tumor-microenvironment interactions on growth, drug permeability to cross blood brain barrier and drug sensitivity. Furthermore, in vivo evaluation of blood brain barrier efficacy of potential active agents will not be possible. Thus, future studies of orthotopic implantation are warranted and currently under development. In the future, it is our hope that efficacious and safe therapies will be discovered and ultimately provide rationale for earlier intervention with targeted therapy, at a time before massive progression of disease and before deterioration of the patient’s overall health. This characterized PDX model provides an opportunity to evaluate and tailor patient specific management in the context of personalized therapy for tumors that are resistant to MEK inhibitors.

## Materials and methods

### Generation of PDX model (PDX RHT-128), ethical considerations, and informed consent

The studies presented were conducted in accordance with the Declaration of Helsinki, and after approval by the Indiana University Institutional Review Board (IRB 1501467439) and obtaining written consent from the subject. All animal studies were carried out in accordance with the Basel Declaration and after the approval of the guideline and regulations of the Indiana University Institutional Animal Care and Use Committee (IACUC #IU-20139-PI: Pollok) and is reported in accordance with ARRIVE guidelines. Fresh tissues from 11 years old male pediatric patient diagnosed with anaplastic pleomorphic xanthoastrocytoma (APXA) was collected at the time of third surgery at Riley Children Hospital, Indianapolis, Indiana. At the time of surgery leptomeningeal spread to was detected. Written informed consent was obtained from the legal guardian of the minor patient and documented in the electronic medical record. Nonnecrotic areas of tumor fragment from the APXA patient were sectioned into approximately 4 mm^3^ pieces. Fragments of the tumor containing both malignant cells and supportive stromal components were implanted subcutaneously into the flanks of anaesthetized (isoflurane 3–3.5%, flowrate 1L/minute during induction, and 0.5L/minute during procedure) 5-week to 8-week-old male NSG mice (Indiana University-Preclinical Modeling and Therapeutic Core, IU-PMTC). During the engraftment phase, tumors were allowed to establish and grow and then were harvested upon reaching a size of 1,500 mm^3^. Harvested tumor was divided for three purposes: (i) subsequent expansion through serial passaging in NSG mice; (ii) biological assays for histologic and molecular characterization of established PDX, (iii) pharmacological study to evaluate drug response in vivo. We have tested the feasibility to freeze/thaw PDX fragments from liquid nitrogen. We have now done this multiple times and the tumor take frequency coming out of liquid nitrogen is at least ~ 95%. The mice (P1 generation) were maintained under pathogen-free conditions and a 12-h light/dark cycle. When P1 tumors reached an approximate size of 1,500 mm^3^, they were harvested, fragmented, and reimplanted into additional mice (P2 generation) while maintained as a live bank according to approved IU Institutional Animal Care and Use Committee protocols. Serial expansion was conducted and tumor from mouse > P3 was used to assess MEK inhibitor response in PDX xenograft model. Tumor pieces from PDX was divided into three mm^2^ pieces and implanted into 20 mice. After tumors reached ~ 200 mm^3^ the mice were randomized into treatment groups (vehicle- 0.5% hydroxypropyl methylcellulose, 0.2% Tween-80 and MEK inhibitor, mirdametinib 10 mg/kg, oral gavage, 5 days on 2 days off). Tumor growth kinetics were monitored in a blinded fashion using an electronic caliper linked to Studylog software. Mice were euthanized once tumors reached a maximum volume of 2000 mm^3^.

### Patient-derived xenoline X-128 and cell cultures

Patient tumor pieces (∼1 mm^2^) were placed in a 10 cm cultured dish and dissociated with trypsin/EDTA for 10 min in 37 °C and CO_2_ 5%. Protein digestion was stopped with supplemented Dulbecco’s Modified Eagle Medium (DMEM) high glucose media (10% FBS). Adherent cells were a mixed population of tumor cells and fibroblasts. Medium was changed every day. These cells were cultivated until approximately 80% confluent. Serial passaging of these heterogeneous cultures was performed, until a homogeneous monolayer of xenolines was present. Xenoline was maintained in Iscove’s Modified Dulbecco’s Medium (IMDM) from Gibco (12440-053) with 10% FBS. Normal human astrocyte was obtained from LONZA (CC-2565) and cultured with AGM™ Astrocyte Growth Medium BulletKit™ (CC-3186). All cultures were maintained at 37° and 5% CO_2_.

### Whole exome sequencing (WES)

WES was used to identify somatic single-nucleotide variations (SNVs) and short insertions/deletions (indels) and copy number variations (CNV) in both patient tumor and PDX.

Exome-captured DNA by Agilent SureSelect Human All Exome was used to prepare libraries and 150-bp paired-end sequencing was performed using an Illumina HiSeq2000 platform (Illumina, USA) by GENEWIZ. Quality control of sequencing data was performed with FastQC. Sequence reads were trimmed to remove possible adapter sequences and nucleotides with poor quality using Trimmomatic v.0.38. Sequence reads were trimmed to remove possible adapter sequences and nucleotides with poor quality using Trimmomatic v.0.38. Mouse read contamination (7.52%) was identified filtered out by implementing a splice-aware global aligner BBSplit program from BBTools suite. This level of mouse contamination is within the range of our non-central nervous system PDX archives^60^″. Burrows-Wheeler Aligner (BWA-MEM)^48,49^ was used to map the reads of each sample to the reference genome. Picard tools were used to sort the SAM files and mark duplicates. Samtools was used to obtain BAM file. Variants were called with Mutec2. GATK was used to perform hard filter SNPs hard-filtering criteria: QualByDepth (QD) 3.0, FisherStrand (FS) > 60.0, RMSMappingQuality (MQ) < − 12.5 and ReadPosRankSum Test (ReadPosRankSum) < − 8.0. Indels hard-filtering criteria: QualByDepth (QD) 200.0 and ReadPosRankSum Test (ReadPosRankSum) < − 20.0. CNV was identified with CNVpytor^61^. Genome landscape was visualized by freely available VCFtoolz, and CNVpytor.

### RNA-seq

To identify changes in gene expression, we used RNA-sequencing. A cDNA library was generated and 150 bp paired-end sequencing was performed using Illumina HiSeq2000 (Illumina, USA) by GENEWIZ. Sequence reads were trimmed to remove possible adapter sequences and nucleotides with poor quality using Trimmomatic v.0.36. The trimmed reads were mapped to the human GRCh38/h38 reference genome available on ENSEMBL using the HISAT2. BAM files were generated because of this step. Feature counts was used to obtain unique hits counts. Biomart was used to map gene ID into Ensemble ID. Differential expression was determined with DESeq2. Exploratory data analysis was implemented to perform hierarchical clustering, GSEA and MsigDB were used to identify overrepresented biological functions of DEG between samples and plot with R package ggplot2 and tidyverse.

### Fusion validation by RT_PCR

Total RNA was extracted by TRIzol (Invitrogen) according to manufactures instructions. Two micrograms of RNA were used to perform cDNA synthesis by iScript cDNA Synthesis Kit (Bio-Rad) and then subjected to PCR reactions. To detect hybrid transcripts the resulting cDNA was subject to amplification with the CDC42SE2 exon 3 primer and BRAF exon 11 primer, which vas visualized by IGV genome browser. In these analyses, all reverse-transcribed samples gave a GAPDH PCR product of the expected size. The amplification conditions were 93 °C for 20 s, 58 °C for 40 s, and 72 °C for 40 s for 35 cycles in a final volume of 25 μL. The products were separated by electrophoresis in agarose gels followed by staining with ethidium bromide.

### Histology/IHC

Mice were sacrificed by CO_2_ asphyxiation at defined time points. Collected tumor tissue was fixed in 10% buffered formalin overnight followed by an additional 24 h in 70% ethanol. Formalin-fixed embedded tissue was cut using microtome with 10-μm thickness. Tissue slides were dried overnight and subjected to deparaffinization in xylene and subjected to H&E and trichrome mason staining.

### Reverse phase protein array (RPPA) analysis of formalin fixed paraffin-embedded (FFPE) xenograft samples

Elaborate methods of this assay have been previously published^62–66^. Briefly, sample preparation can be divided into three main steps:

#### Protein lysate generation from PDX FFPE

PDX FFPE blocks were sectioned at 8 μm onto glass slides, continued with the sectioned deparaffinization in xylene (2–6 section per samples), followed by rehydration in 100% and 95% ethanol. Next, tissue slides were briefly fixed in 70% ethanol containing protease inhibitors (Complete Mini EDTA-free, Roche, Indianapolis, IN, USA), then dehydrated in the series of 95% ethanol, 100% ethanol. Further, sample was lysed directly from slides in an appropriate volume of extraction buffer containing 50% Tissue Protein Extraction Reagent (T-PER, Thermo Fisher Scientific; Waltham, MA, USA), 47.5% 2 × Tris–Glycine SDS sample buffer (Invitrogen), and 2.5% β-mercaptoethanol (Thermo Fisher Scientific; Waltham, MA, USA). The resulting whole tissue lysates were heated for 8 min at 100 °C. Sample lysates was stored at − 80 °C. Prior to lysate printing, samples were reheated at 100 °C for 2 min, vortexed and briefly centrifuged.

#### Array printing and analysis

First, the total protein concentration in each sample was estimated by printing onto a nitrocellulose slide (Avid, Grace Biolabs, Bend, OR, USA) along with a BSA standard concentration curve using a Quanterix 2470 Arrayer equipped with 180um pins at a deposition volume of ~ 11nL per sample. Next, printed sample was stained by Sypro Ruby Protein Blot Stain (Invitrogen; Waltham, MA, USA) according to manufacturer’s instructions to estimate total protein levels, and tissue lysate concentrations were adjusted to a maximum of 250ug/ml with extraction buffer. The same printing condition was used for RPPA printing, followed by antibody staining and image analysis as previously described^35–38^ . Briefly, arrays were stained with 129 antibodies against signaling endpoints, mainly phosphorylated and cleaved protein products to investigate activated signaling pathway. Antibody specificity was confirmed by western blot prior of RPPA analysis. MicroVigene v5 Software Package (Vigenetech; Carlisle, MA, USA) was used to assess antibody staining’s intensities^62,64,65^.

#### RPPA statistical analysis

Each data point represents the average of 3 replicate printed spots. Next, normalization to total protein following the subtraction of negative control was done for each data point. To ensure data reliability we prioritized RPPA data where coefficient of variation (CV) among the replicate spots were ≤ 10%^39^. Additionally, all data values within a group, such as a specific kinase, can be compared to the same data point in other group, but data values within a group such as different kinases or signaling proteins cannot be directly compared. If zero value is obtained then those are true zero values where spot intensities were either not above local background or not above negative control spot intensities^66^.

### Methylene blue cell proliferation assay

Xenoline X-128 (10,000 cells/well) were seeded in 96-well plates and incubated for 24 h at 37 °C and 5% CO_2_. The following day, cells were treated with drugs with defined concentrations. All drugs for in vitro study. Trametinib, mirdametinib and Everolimus were dissolved in DMSO for the preparation of stock solutions (10 mmol/L). Cell viability was determined by Methylene Blue assay. 100 μL of methanol was added to stained plate to dissolve the dye crystals, and the plates were incubated for a further 15 min at room temperature before the absorbance was measured at *λ* = 660 nm using a Micro Plate Reader (BioTek Synergy HTX).

## Supplementary Information

Supplementary Information.

## Data Availability

The datasets generated during and/or analyzed during the current study are available in the Gene Expression Omnibus GEO Repository (accession number GSE213162, https://www.ncbi.nlm.nih.gov/geo/query/acc.cgi?acc=GSE213162). Models generated from this work are available upon request to corresponding authors and MTA agreement to Indiana University.
