# Supplementary material for: Establishment and characterization of patient-derived xenograft of a rare pediatric anaplastic pleomorphic xanthoastrocytoma (PXA) bearing a CDC42SE2-BRAF fusion
Source: Sci Rep. 2023 Jun 6;13:9163. doi: 10.1038/s41598-023-36107-2 (PMC10244396; doi:10.1038/s41598-023-36107-2)
Supplement: Supplementary file 1 — Supplementary Information. [file 41598_2023_36107_MOESM1_ESM.pdf]

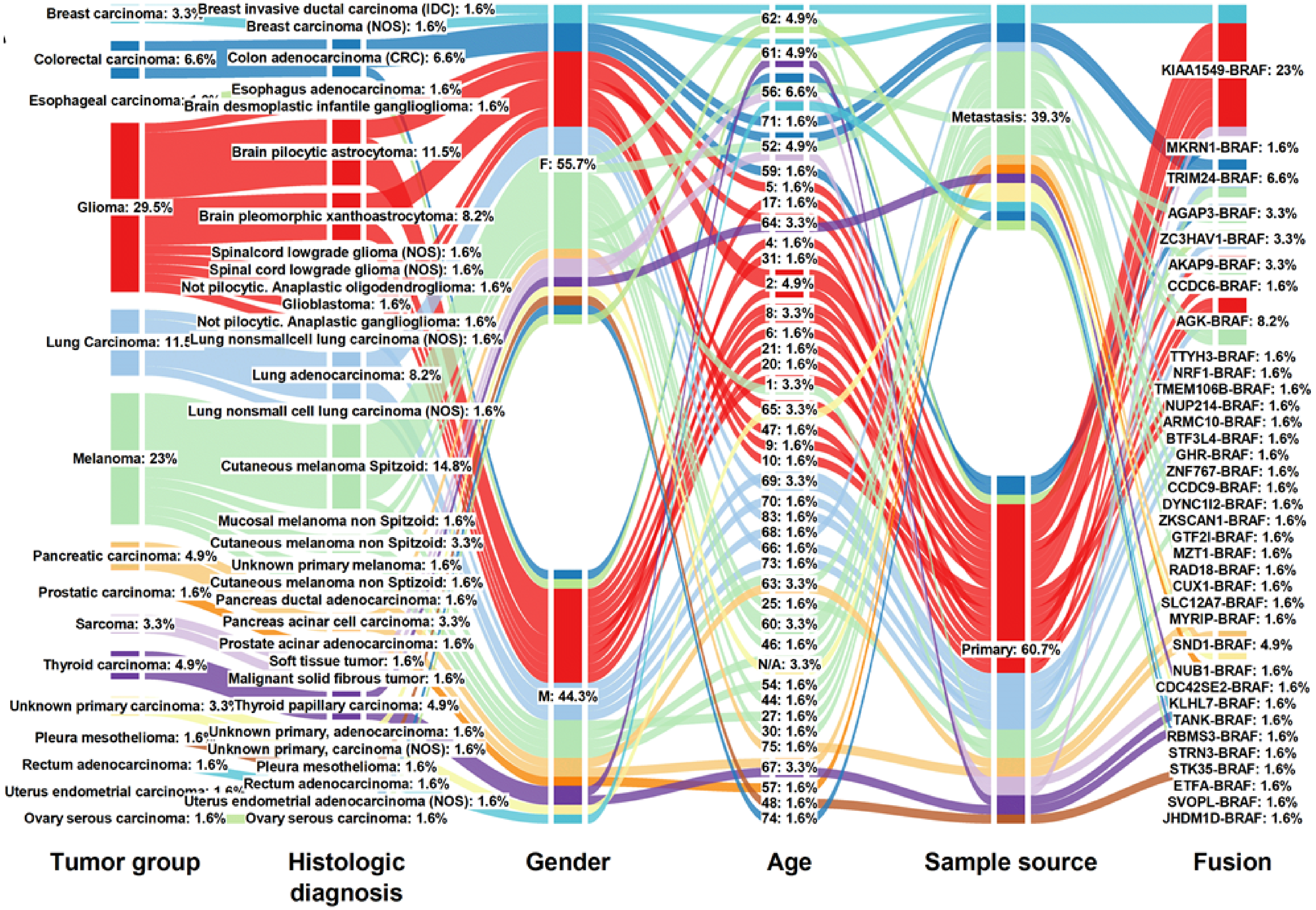

Supplementary Figure S1. Differential BRAF fusion partner across solid tumors. Figure generated by OriginPro 2023. https://www.originlab.com/

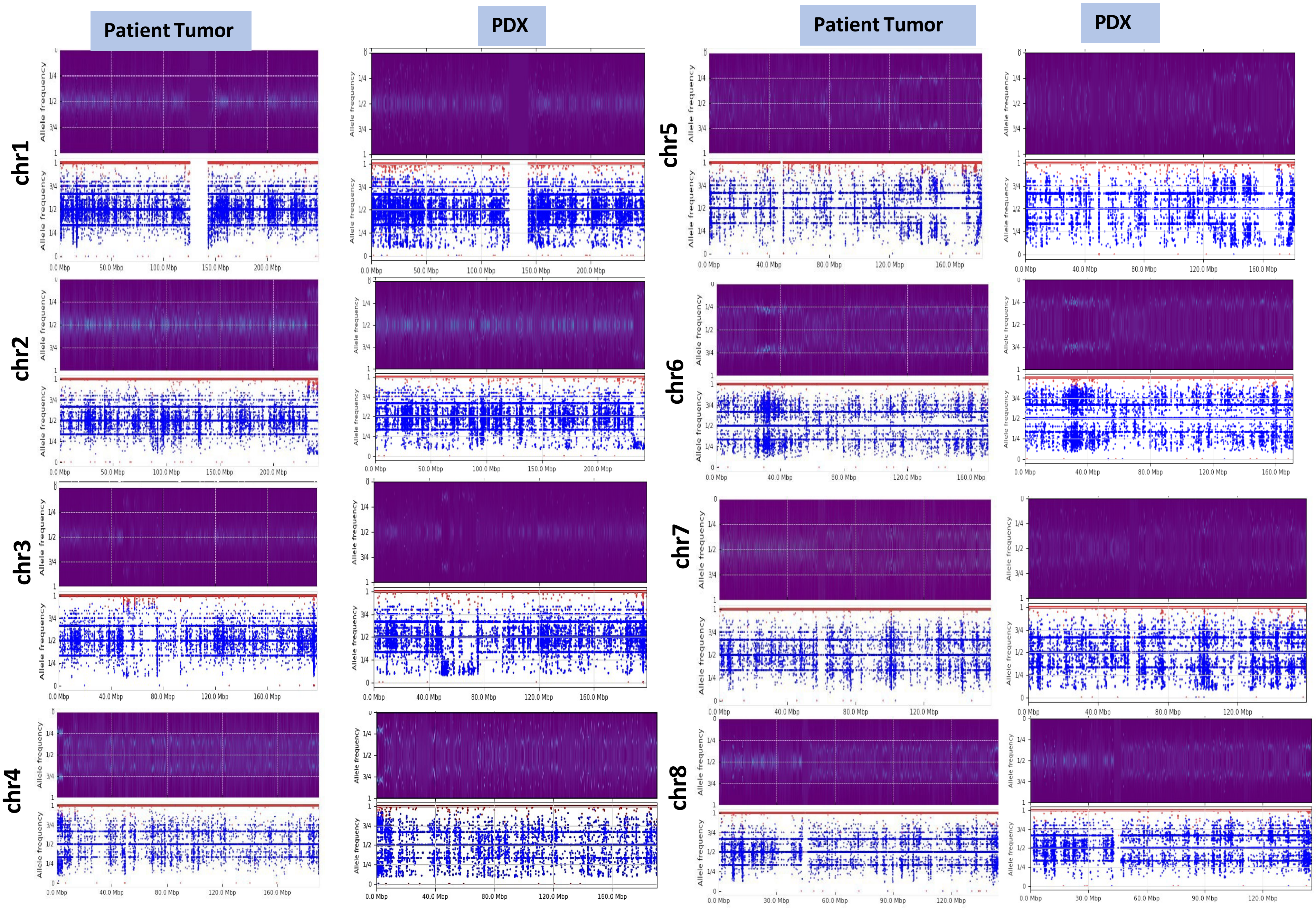

Supplementary Figure S2A. Chromosomal copy number variations (CNV) of chr 1-chr 8 in patient tumor are reflected in PDX model (Cont').

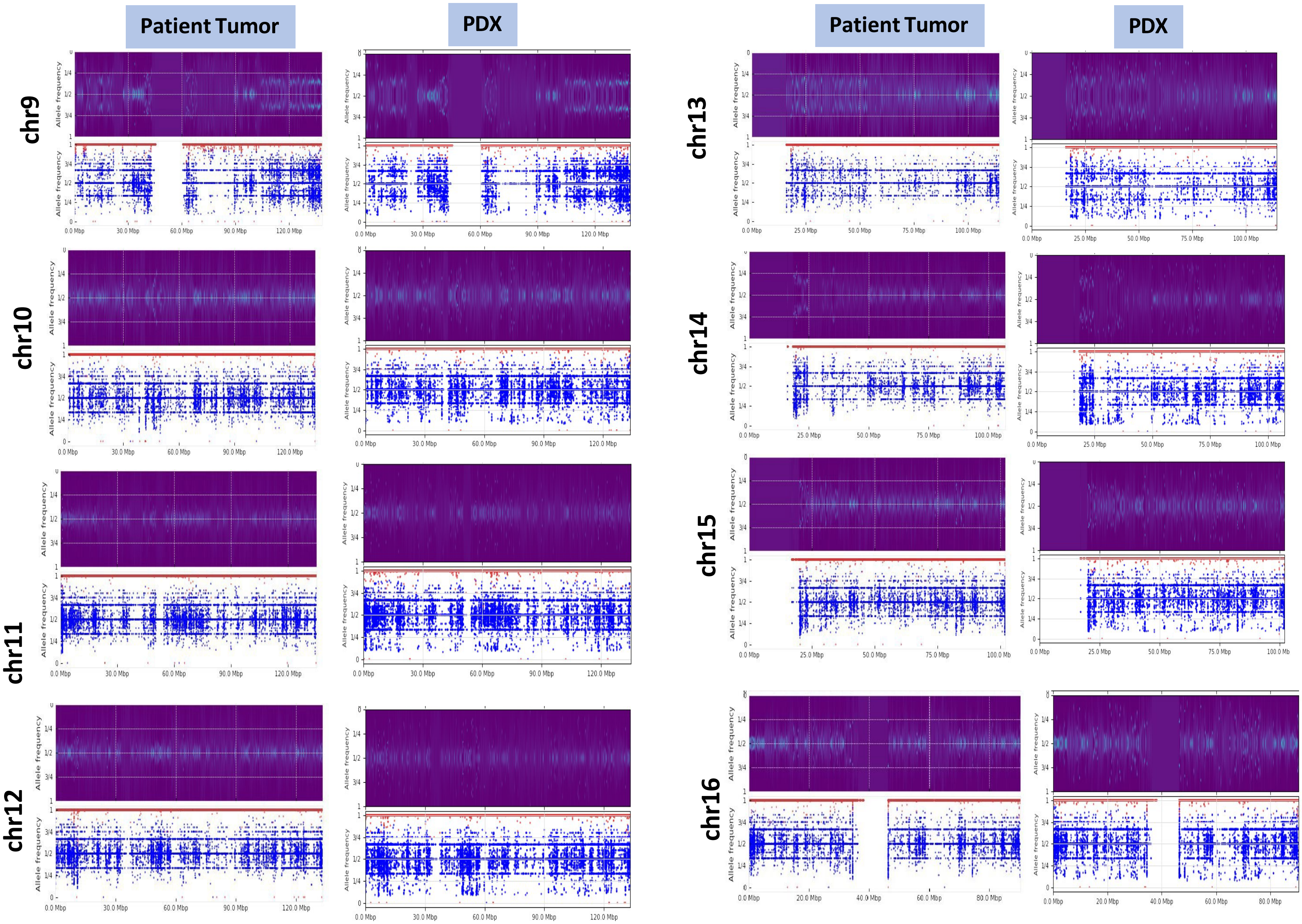

Supplementary Figure S2B. Chromosomal copy number variations (CNV) of chr 9-chr 16 in patient tumor are reflected in PDX model (Cont').

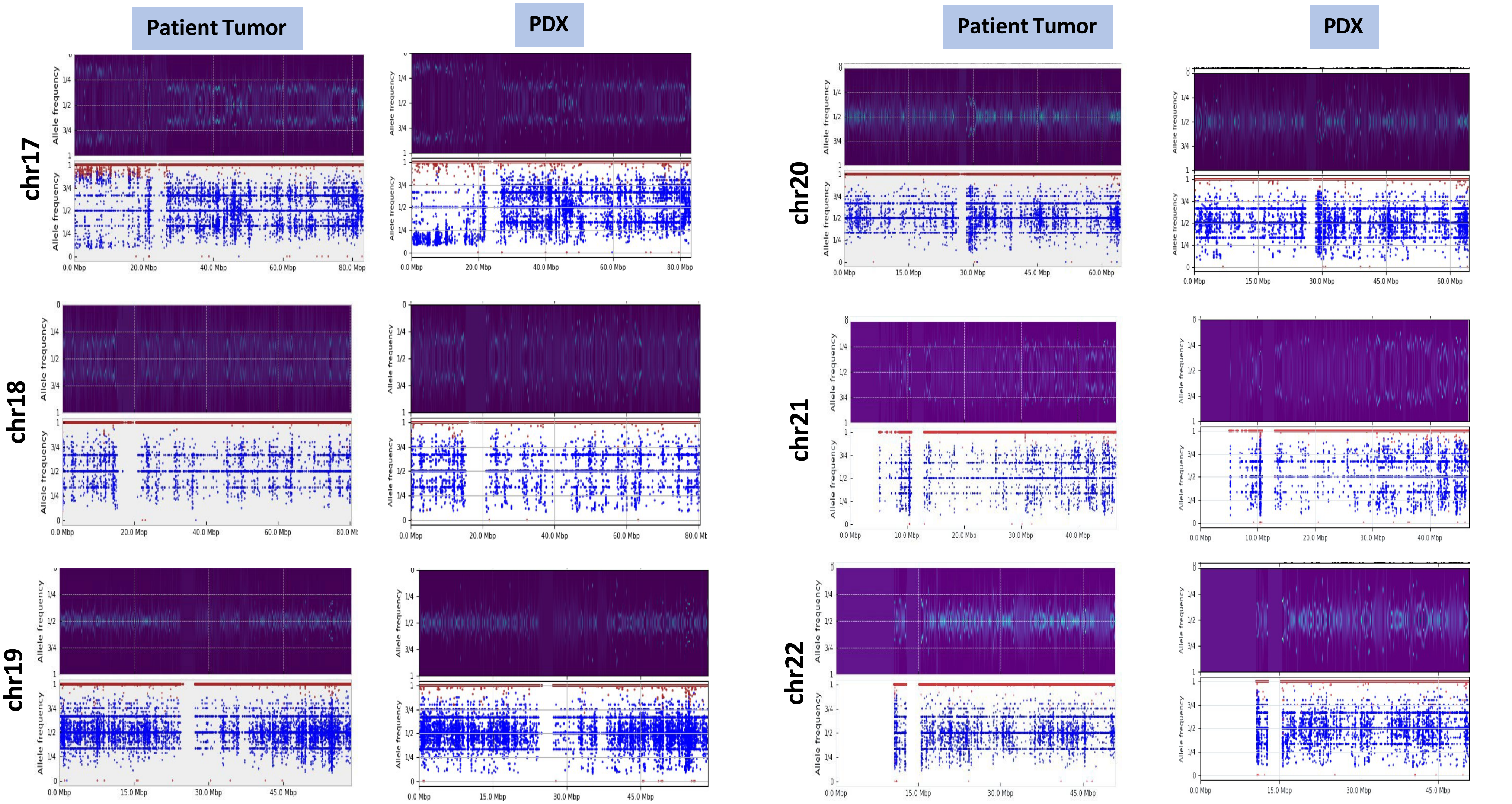

Supplementary Figure S2C. Chromosomal copy number variations (CNV) of chr 9-chr 16 in patient tumor are reflected in PDX model (Cont').

Supplementary Figure S3. RT-PCR

RT-PCR

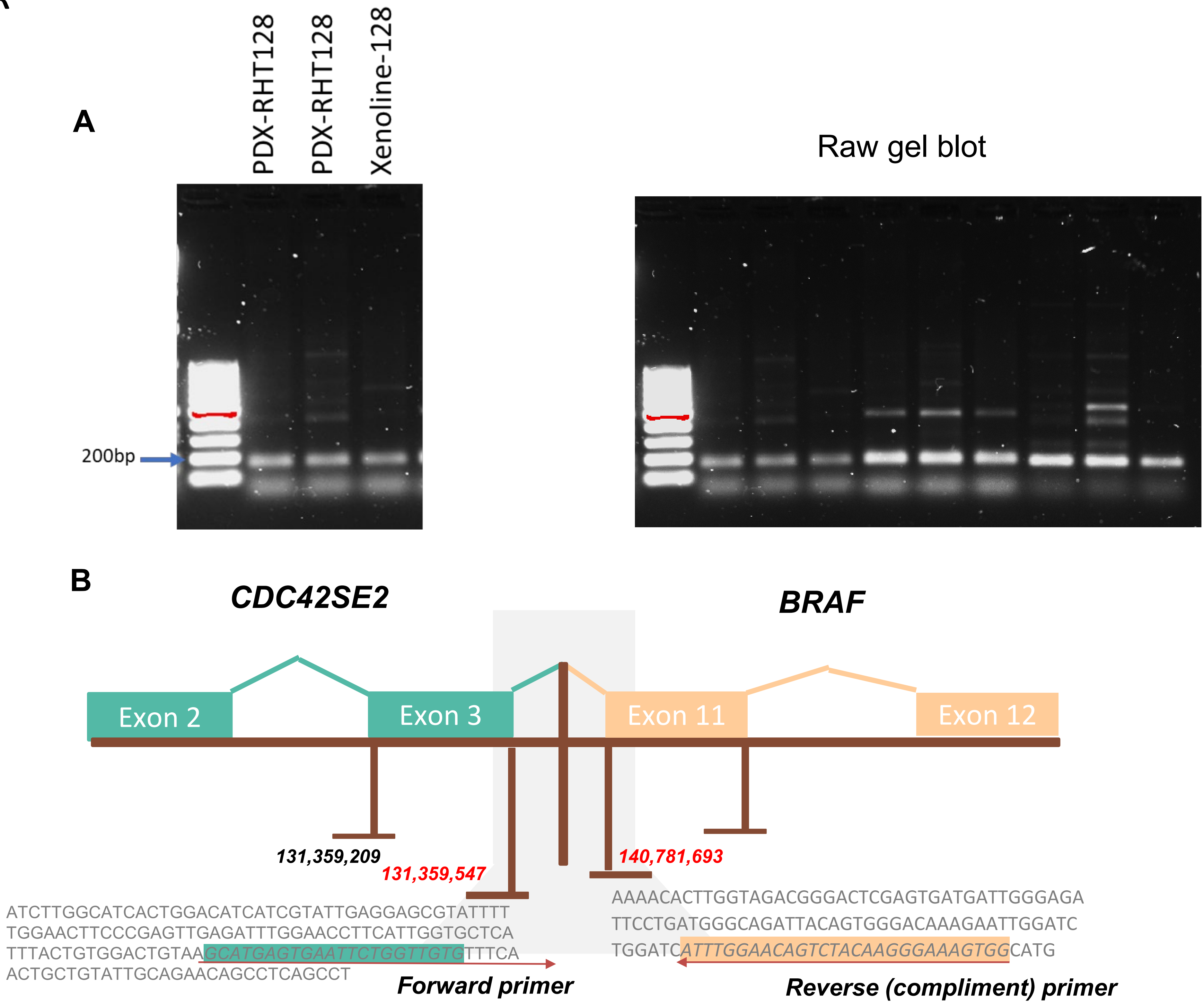

Supplementary Figure S3. Fusion CDC42SE2 BRAF is expressed in transcript as shown with (A) RT PCR with band of 200 bp (left) from raw gel image (right) using **(B)** primer design based on fusion breakpoint.

Supplementary Table S1
Meta genes k-means cluster A

Cluster	Genes Ensembl ID	Gene ID
A	ENSG00000122641	INHBA
A	ENSG00000096696	DSP
A	ENSG00000113083	LOX
A	ENSG00000164484	TMEM200A
A	ENSG00000115414	FN1
A	ENSG00000108821	COL1A1
A	ENSG00000107984	DKK1
A	ENSG00000197614	MFAP5
A	ENSG00000164692	COL1A2
A	ENSG00000198959	HEL-S-45
A	ENSG00000113389	NPR3
A	ENSG00000182492	BGN
A	ENSG00000073756	PTGS2
A	ENSG00000105976	MET
A	ENSG00000113739	STC2
A	ENSG00000120708	TGFBI
A	ENSG00000147883	CDKN2B
A	ENSG00000137801	THBS1
A	ENSG00000164176	EDIL3
A	ENSG00000135480	KRT7
A	ENSG00000170955	CAVIN3
A	ENSG00000106366	SERPINE1
A	ENSG00000164161	HHIP
A	ENSG00000115648	MLPH
A	ENSG00000072682	P4HA2
A	ENSG00000147889	CDKN2A
A	ENSG00000133816	MICAL2
A	ENSG00000196611	MMP1
A	ENSG00000148677	ANKRD1

Supplementary Table S2
Meta genes k-means cluster B

Cluster	Genes Ensembl ID	Gene ID
B	ENSG00000277586	NEFL
B	ENSG00000184012	TMPRSS2
B	ENSG00000134595	SOX3
B	ENSG00000214285	NPS
B	ENSG00000167281	RBFOX3
B	ENSG00000213235	EEF1A1P16
B	ENSG00000187955	COL14A1
B	ENSG00000143631	FLG
B	ENSG00000174600	CMKLR1
B	ENSG00000104722	NEFM
B	ENSG00000185046	ANKS1B
B	ENSG00000115353	TACR1
B	ENSG00000142619	PADI3
B	ENSG00000238266	LINC00707
B	ENSG00000164616	FBXL21P
B	ENSG00000156675	RAB11FIP1
B	ENSG00000204434	POTEKP
B	ENSG00000265962	GACAT2
B	ENSG00000142515	KLK3
B	ENSG00000180287	PLD5
B	ENSG00000144681	STAC
B	ENSG00000089199	CHGB
B	ENSG00000257907	EEF1A1P17
B	ENSG00000179131	
B	ENSG00000137673	MMP7
B	ENSG00000187653	TMSB4XP8
B	ENSG00000185559	DLK1
B	ENSG00000226986	PRELID1P5
B	ENSG00000230593	PPIAP40

Supplementary Table S3
Meta genes k-means cluster C

Cluster	Genes Ensembl ID	Gene ID
C	ENSG00000150893	FREM2
C	ENSG00000133048	CHI3L1
C	ENSG00000182132	KCNIP1
C	ENSG00000184486	POU3F2
C	ENSG00000179761	PIPOX
C	ENSG00000262655	SPON1
C	ENSG00000166501	PRKCB
C	ENSG00000104327	CALB1
C	ENSG00000170439	METTL7B
C	ENSG00000123496	IL13RA2
C	ENSG00000182175	RGMA
C	ENSG00000237515	SHISA9
C	ENSG00000075340	ADD2
C	ENSG00000075461	CACNG4
C	ENSG00000108018	SORCS1
C	ENSG00000118946	PCDH17
C	ENSG00000258754	LINC01579
C	ENSG00000253967	
C	ENSG00000260664	
C	ENSG00000120093	HOXB3
C	ENSG00000007171	NOS2
C	ENSG00000103449	SALL1
C	ENSG00000106236	NPTX2
C	ENSG00000186466	AQP7P1
C	ENSG00000171385	KCND3
C	ENSG00000197444	OGDHL
C	ENSG00000179344	HLA-DQB1
C	ENSG00000185686	PRAME
C	ENSG00000211829	TRDC

Supplementary Table S4
Meta genes k-means cluster D

Cluster	Genes Ensembl ID	Gene ID
D	ENSG00000090104	RGS1
D	ENSG00000275395	FCGBP
D	ENSG00000170049	KCNAB3
D	ENSG00000101198	NKAIN4
D	ENSG00000173369	C1QB
D	ENSG00000164106	SCRG1
D	ENSG00000130600	H19
D	ENSG00000110077	MS4A6A
D	ENSG00000159189	C1QC
D	ENSG00000080493	SLC4A4
D	ENSG00000135439	CENTG1
D	ENSG00000174672	BRSK2
D	ENSG00000196735	HLA-DQA1
D	ENSG00000173372	C1QA
D	ENSG00000162946	DISC1
D	ENSG00000099954	CECR2
D	ENSG00000177575	CD163
D	ENSG00000084636	COL16A1
D	ENSG00000010327	STAB1
D	ENSG00000114841	DNAH1
D	ENSG00000069493	CLEC2D
D	ENSG00000250067	YJEFN3
D	ENSG00000214548	MEG3
D	ENSG00000137841	PLCB2
D	ENSG00000204248	COL11A2
D	ENSG00000180353	HCLS1
D	ENSG00000128564	VGF
D	ENSG00000182272	B4GALNT4
D	ENSG00000170390	DCLK2

Supplementary Table S5
Meta genes k-means cluster E

Cluster	Genes Ensembl ID	Gene ID
E	ENSG00000125462	MIR9-1HG
E	ENSG00000101203	COL20A1
E	ENSG00000181790	ADGRB1
E	ENSG00000163873	GRIK3
E	ENSG00000112280	COL9A1
E	ENSG00000130287	NCAN
E	ENSG00000150625	GPM6A
E	ENSG00000089169	RPH3A
E	ENSG00000184221	OLIG1
E	ENSG00000144285	SCN1A
E	ENSG00000187398	LUZP2
E	ENSG00000104112	SCG3
E	ENSG00000182103	FAM181B
E	ENSG00000131095	GFAP
E	ENSG00000132692	BCAN
E	ENSG00000167614	TTYH1
E	ENSG00000150275	PCDH15
E	ENSG00000147588	PMP2
E	ENSG00000077522	ACTN2
E	ENSG00000196338	NLGN3
E	ENSG00000177807	KCNJ10
E	ENSG00000136160	EDNRB
E	ENSG00000128683	GAD1
E	ENSG00000205927	OLIG2
E	ENSG00000109956	B3GAT1
E	ENSG00000101144	BMP7
E	ENSG00000120251	GRIA2
E	ENSG00000123560	PLP1
E	ENSG00000101104	PABPC1L

Supplementary Figure S4. Overlap enriched pathway from multiomics analysis

DNA
Bladder cancer
Graft-versus-host disease
Melanoma
Long-term depression
Chronic myeloid leukemia
Antigen processing and presentation
Longevity regulating pathway
GnRH signaling pathway
Natural killer cell mediated cytotoxicity
Hepatocellular carcinoma
Signaling pathways regulating pluripotency of stem cells
Human T-cell leukemia virus 1 infection
Gastric cancer
Hepatitis B
Epstein-Barr virus infection
Viral carcinogenesis

Protein
Acute myeloid leukemia
ErbB signaling pathway
Central carbon metabolism in cancer
PD-L1 expression and PD-1 checkpoint pathway in cancer
Choline metabolism in cancer
Th17 cell differentiation
HIF-1 signaling pathway
Insulin signaling pathway
Growth hormone synthesis, secretion and action
Thyroid hormone signaling pathway
Phospholipase D signaling pathway
Hepatitis C
Human cytomegalovirus infection
Kaposi sarcoma-associated herpesvirus infection
Chemical carcinogenesis
Shigellosis
Coronavirus disease

DNA U Protein
Glioma
Endocrine resistance
Pancreatic cancer
Non-small cell lung cancer
Prostate cancer
Cellular senescence
FoxO signaling pathway
Colorectal cancer

RNA
Amino sugar and nucleotide sugar metabolism
Protein export
Biosynthesis of nucleotide sugars
DNA replication
Axon guidance
P53 signaling pathway
ECM-receptor interaction
AGE-RAGE signaling pathway in diabetic complications
Small cell lung cancer
Bacterial invasion of epithelial cells
TGF-beta signaling pathway
Protein processing in endoplasmic reticulum
Focal adhesion
Herpes simplex virus 1 infection
Rap1 signaling pathway
Regulation of actin cytoskeleton
Endocytosis
Hippo signaling pathway
PI3K-Akt signaling pathway
Ras signaling pathway
Salmonella infection
Metabolic pathways

DNA U Protein
Glioma
Endocrine resistance
Pancreatic cancer
Non-small cell lung cancer
Prostate cancer
Cellular senescence
FoxO signaling pathway
Colorectal cancer

DNA u RNA
Cell cycle
Transcriptional misregulation in cancer
MAPK signaling pathway

RNA U Protein
MicroRNAs in cancer
Human papillomavirus infection

DNA U RNA U Protein
EGFR tyrosine kinase inhibitor resistance
Proteoglycans in cancer
Pathways in cancer

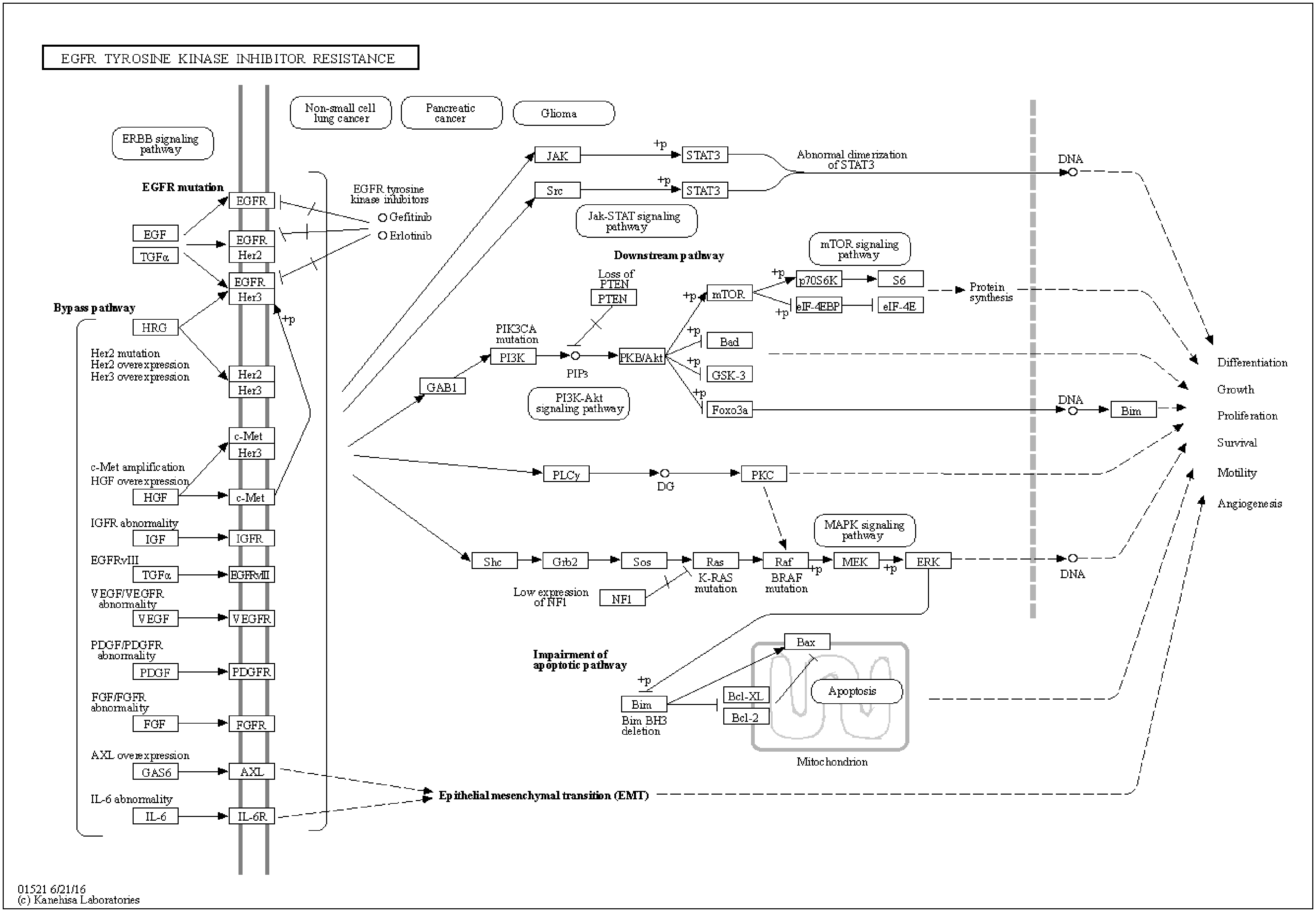

Supplementary Figure S5A. Enriched KEGG EGFR Tyrosine Kinase inhibitor pathway in PDX and corresponding patient tumor.

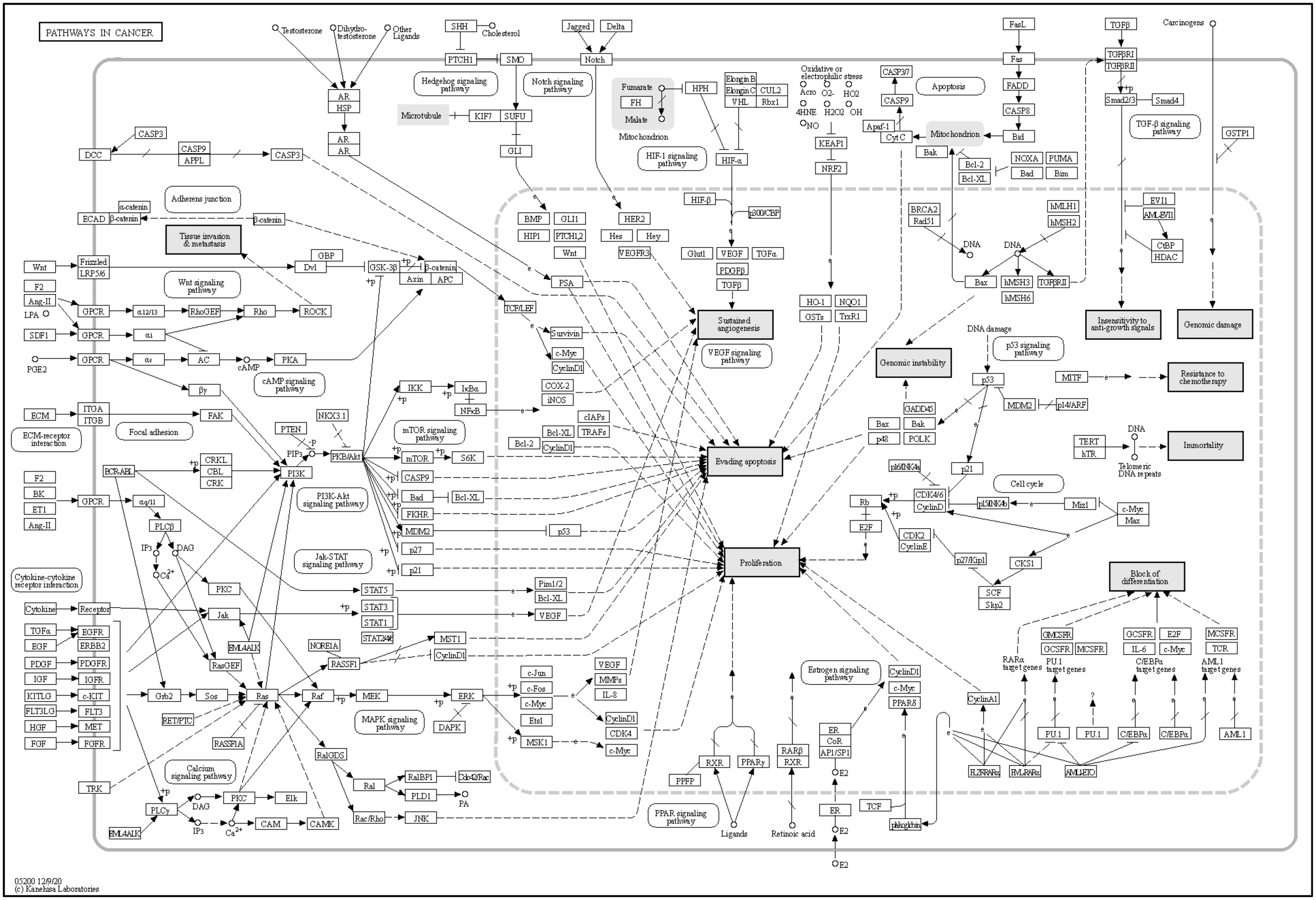

Supplementary Figure S5B. Enriched pathways in cancer in PDX and corresponding patient tumor.

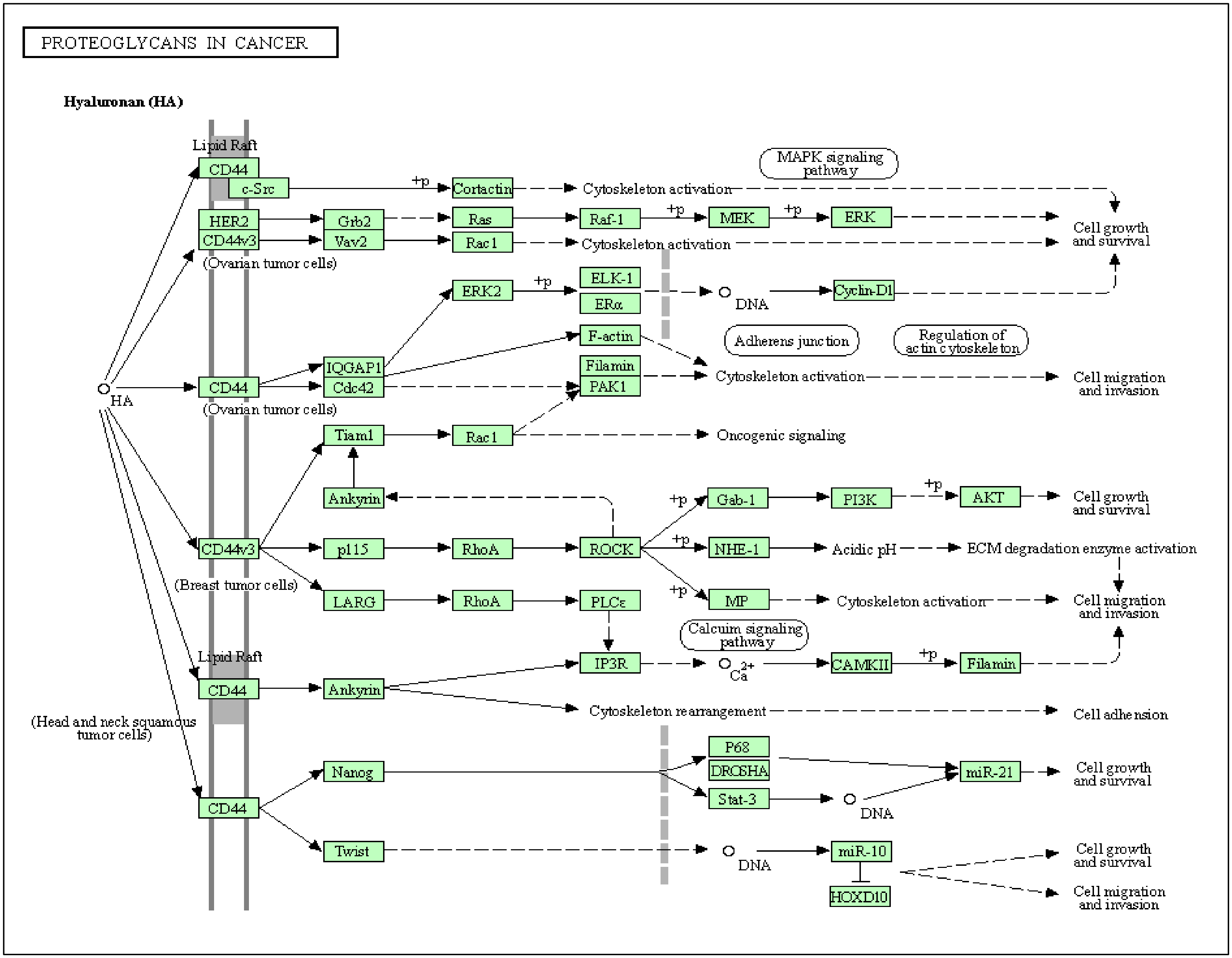

Supplementary Figure S5C. Enriched proteoglycan pathway in PDX and corresponding patient tumor.
